# Interfacial Interactions of Nanoparticles and Molecular Nanostructures with Model Membrane Systems: Mechanisms, Methods, and Applications

**DOI:** 10.3390/membranes16040134

**Published:** 2026-04-01

**Authors:** Konstantin Balashev

**Affiliations:** Laboratory of Biophysical Chemistry, Department of Physical Chemistry, Faculty of Chemistry and Pharmacy, Sofia University, 1, James Bourchier Ave., 1164 Sofia, Bulgaria; fhkb@chem.uni-sofia.bg

**Keywords:** nano-bio interface, model membrane systems, Langmuir monolayers, supported lipid bilayers (SLBs), liposomes, Brewster Angle Microscopy (BAM), atomic force microscopy (AFM), molecular dynamics (MD) simulations, drug and gene delivery, nanotoxicology

## Abstract

This review surveys how nanoparticles and biomolecular nanosized structures interact with model membrane systems, and how these interfacial processes govern their performance in drug and gene delivery, antimicrobial strategies, biosensing, and nanotoxicology. The nanostructures covered include polymeric nanoparticles, lipid-based carriers, peptide nanostructures, dendrimers, and multifunctional hybrids. Model membranes span Langmuir monolayers, supported lipid bilayers, vesicles/liposomes across sizes, and emerging hybrid or asymmetric constructs that better approximate native complexity. Mechanistically, interactions follow recurrent routes—surface adsorption, bilayer insertion, pore formation, and lipid extraction/reorganization—regulated by particle size, morphology, charge, ligand architecture, and lipophilicity, in conjunction with membrane composition, phase state, curvature, and asymmetry. A multiscale toolkit links structure, mechanics, and dynamics: Langmuir troughs and Brewster Angle Microscopy map thermodynamics and mesoscale morphology; atomic force microscopy and quartz crystal microbalance with dissipation resolve nanoscale topography and viscoelasticity; fluorescence microscopy/spectroscopy reports on localization and packing; neutron and X-ray reflectometry quantify vertical structure; molecular dynamics provides atomistic pathways and design hypotheses. Historically, the field advanced from early monolayers and bilayers, through the fluid mosaic model, to raft microdomains and modern biomimetic systems, enabling increasingly realistic experiments. Key advances include cross-method integration linking experimental observations with image-based computational models; persistent debates concern the translation from simplified models to living membranes, the role of dynamic coronas, and scale/force-field limits in simulations. Future efforts should prioritize hybrid models incorporating proteins and asymmetric lipidomes, standardized reporting and reference systems, rigorous coupling of experiments with calibrated simulations and machine learning, and alignment with safety-by-design and regulatory expectations, thereby shifting interfacial measurements from descriptive observation to predictive design rules.

## 1. Introduction

The exploration of nano–bio interfaces is fundamental to the development of advanced drug delivery systems, the refinement of diagnostic techniques, and the assessment of nanotoxicological risks inherent in biomedical applications [1,2]. Biological membranes, whether at the cell surface or within organelles, serve as the primary interaction sites for nanomaterials within living systems. Consequently, a thorough understanding of these interfacial processes is paramount for enhancing therapeutic efficacy while simultaneously minimizing unintended toxicity [3]. Recent investigations, both experimental and theoretical, have yielded significant insights into nanoparticle–membrane interactions, underscoring their intrinsic complexity and emphasizing the necessity for more sophisticated experimental models and analytical methods capable of bridging the gap between simplified systems and the dynamic, heterogeneous nature of living cells [4,5,6,7]. Comprehensive surveys of nanoparticle–bio-membrane interactions have further unified diverse biophysical mechanisms across living and synthetic membrane systems, illuminating the roles of membrane composition, nanoparticle morphology, and interfacial forces in governing interaction outcomes [8].

The nexus between nanomaterials and biological entities, termed the nano–bio interface, holds significant relevance across three primary biomedical fields: therapeutic delivery, diagnostics, and the assessment of nanotoxicological impacts [4,9]. These concepts have been reiterated in state-of-the-art reviews highlighting the need to integrate experimental, theoretical, and simulation insights for a comprehensive understanding of nano–bio interface phenomena [8]. At the therapeutic frontier, nanoparticles leverage their nanoscale dimensions, extensive surface area, and adjustable physicochemical attributes to facilitate enhanced targeted delivery, modulated release kinetics, and multifaceted functionalities [9,10]. In the context of targeted delivery, these nano–bio interfaces enable selective interaction with specific cell types, thereby augmenting therapeutic precision and diminishing systemic toxicity [2,10]. Furthermore, engineered release mechanisms facilitate either sustained drug liberation or stimuli-responsive discharge, optimizing pharmacological outcomes while concurrently mitigating adverse effects [3]. Within multifunctional paradigms, nanoparticles are engineered to integrate both therapeutic and diagnostic capabilities (theranostics), thereby permitting real-time monitoring of treatment progression [10,11].

In the realm of diagnostics, the unique optical, electronic, and surface characteristics of nanomaterials significantly enhance bioimaging modalities and biosensor technologies [11]. Nanoparticles improve imaging contrast and resolution, facilitating early disease detection and sensitive surveillance of therapeutic responses [11]. Similarly, nano–bio interfaces are crucial for the development of ultrasensitive biosensors capable of detecting biomarkers present in low abundance, thereby improving diagnostic precision [11]. Within this framework, computational simulations have emerged as valuable tools for predicting nanoparticle interactions with diagnostic membrane platforms, enabling the rational design of responsive and selective nanocarriers [12]. At the same time, elucidating the mechanisms governing nanoparticle–membrane interactions remains essential for addressing nanotoxicological concerns, including genotoxicity, immune activation, and long-term biocompatibility [5,13]. Collectively, recent reviews emphasize that theoretical and simulation-based approaches are indispensable for resolving mechanistic complexity and achieving quantitative predictions of nanoparticle–membrane energetics, particularly in the contexts of nanotoxicology and nanomedicine design [12].

Despite these advances, many challenges still remain [13]. Biological membranes are structurally and compositionally heterogeneous, shaped by cell type, physiological state, and microenvironment. Their dynamic nature profoundly influences nanoparticle behavior, complicating the extrapolation of data from model systems to living organisms [5]. Thus, comprehensive studies of nano–bio interactions remain essential to guide the safe and effective design of nanomedicines [14].

**Figure 1:**
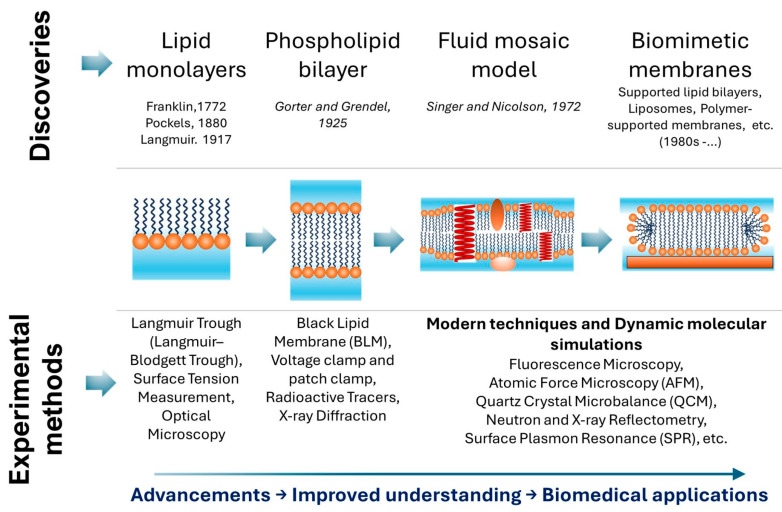
Historical progression and key milestones in biomembrane interface research. Major discoveries—from early lipid monolayers and the phospholipid bilayer model to the fluid mosaic model and modern biomimetic architectures—are shown alongside representative experimental methods. This evolution illustrates the progressive refinement of membrane models and analytical approaches that underpin current biomedical applications. Figure 1 serves as a historical framework; modern self-assembled lipid nanoparticles and hybrid membranes are introduced later in Section 2. Original schematic.

The conceptualization of biological membranes has been significantly influenced by a series of pivotal discoveries that continue to inspire contemporary investigations at the nano–bio interface. Figure 1 summarizes the historical development of biomembrane research, providing context for the more detailed discussion of contemporary membrane models and nanostructures presented later in this work. Early experiments on lipid monolayers by Franklin, Pockels, and Langmuir established the foundation for understanding membrane surface properties [15]. The subsequent proposal of the lipid bilayer model by Gorter and Grendel (1925) provided the first structural framework, later refined by Singer and Nicolson’s fluid mosaic model (1972), which described membranes as dynamic, protein-embedded lipid bilayers [16]. The discovery of lipid rafts and the development of biomimetic systems, including supported lipid bilayers (SLBs) and synthetic asymmetric membranes, further advanced the field by enabling controlled investigations of membrane organization, dynamics, and function [16,17]. These milestones collectively highlight the progressive refinement of models that bridge fundamental principles with biomedical applications [18,19].

To analyze these complex interactions, researchers increasingly rely on model membrane systems that emulate essential features of biological membranes while reducing their complexity [20]. Computational modeling has further expanded this repertoire by introducing validated coarse-grained and multiscale membrane representations capable of capturing mesoscale deformations and lipid heterogeneity [21]. Recent simulation-based reviews highlight the utility of these model systems in resolving lipid packing dynamics, curvature effects, and collective membrane responses at nanoscopic resolution [20]. Langmuir monolayers provide insights into lipid packing and surface interactions; SLBs enable studies of protein binding and membrane-associated signaling [22]; vesicular systems such as giant unilamellar vesicles (GUVs) and small unilamellar vesicles (SUVs) mimic three-dimensional membrane curvature and encapsulation [23]; and plasma membrane–derived vesicles retain natural heterogeneity [23]. More advanced asymmetric and biomimetic models approximate native phospholipid distributions [24], while computational approaches, particularly molecular dynamics simulations, complement experiments by offering molecular-scale resolution of dynamic processes [6,7,24,25,26,27].

While experimental systems have played a central role in advancing this field, computational and theoretical approaches have become equally indispensable for probing the energetics, kinetics, and structural consequences of nanoparticle–membrane interactions. Molecular dynamics simulations, whether performed at all-atom or coarse-grained resolutions, enable time-resolved visualization of lipid rearrangements, nanoparticle adsorption, and membrane disruption processes at nanometer and nanosecond scales [6,26]. Contemporary analyses highlight how advances in coarse-grained and mesoscale simulation techniques provide predictive insight into membrane bending, nanoparticle wrapping, and energetic barriers to translocation. These approaches have been particularly useful for connecting experimental observables—such as phase transitions, insertion pressure, or membrane rigidity—to underlying molecular determinants such as hydrophobic mismatch, electrostatic complementarity, and curvature induction [7,25].

At a broader level, theoretical frameworks derived from membrane biophysics have helped rationalize how nanoparticles perturb lipid assemblies. Concepts rooted in continuum modeling, such as membrane bending energy, elastic deformation, and line tension, provide predictive insight into the free energy penalties associated with nanoparticle insertion, pore nucleation, and lipid extraction [25]. Thermodynamic analyses of lipid packing and lateral pressure profiles have clarified how nanoparticle binding alters phase equilibria and induces transitions between fluid and condensed membrane states [7,22]. Complementary reviews in theoretical membrane biophysics emphasize the value of continuum and statistical mechanical frameworks for predicting key phenomena such as membrane bending energy, line tension, and force-mediated pore formation [28]. Together, these computational and theoretical approaches provide a coherent mechanistic context that complements experimental observations and enables more robust interpretation of structure–function relationships [25,26].

Importantly, multiscale theoretical approaches increasingly highlight the degree to which nanoparticle–membrane interactions are governed by collective behavior rather than isolated molecular events. Cooperative lipid rearrangements, nanoparticle clustering, and membrane-mediated forces all contribute to emergent phenomena that are difficult to capture experimentally but can be revealed through simulation. As a result, computational methods have evolved beyond purely hypothesis-generating tools and now enable systematic exploration of parameter space that remains inaccessible to conventional experimentation [6]. Emerging multiscale and mesoscale strategies seek to bridge molecular simulations with continuum descriptions, thereby addressing the time- and length-scale gaps between microscopic interactions and macroscopic membrane behavior [29]. Taken together, these modeling approaches offer complementary perspectives, although none fully captures the complexity of living membranes [27]. At the same time, computational studies of membrane systems face inherent challenges that continue to shape their interpretation and applicability. Limitations associated with force-field accuracy, accessible time and length scales, and the representation of compositional heterogeneity constrain the direct translation of simulation results to complex biological membranes [6,26]. Bridging molecular simulations with experimental observables remains an active area of development, requiring careful validation and multiscale integration [26]. Recognizing these challenges is essential for placing computational insights into proper context and motivates the combined experimental–theoretical approaches discussed throughout this review. These considerations highlight the need for a coherent framework that connects model membrane systems, experimental methodologies, and multiscale computational approaches.

This review provides a comprehensive synthesis of current knowledge on the interactions between nanoparticles, molecular nanostructures, and model membrane systems. Through a critical evaluation of the advantages and limitations of widely used experimental and computational models, it analyzes the fundamental physicochemical principles governing key interfacial phenomena, including adsorption, insertion, membrane perturbation, pore formation, and cargo transport. Particular emphasis is placed on emerging experimental methodologies and computational approaches that enable multiscale insight into nano–bio interfaces, integrating molecular-level mechanisms with mesoscale and macroscopic membrane responses. By highlighting representative case studies and identifying unresolved challenges, this review seeks to inform the rational design of nanomedicines, biosensors, and diagnostic platforms, while addressing safety and biocompatibility considerations central to nanotoxicology.

While recent reviews have examined nanoparticle–membrane interactions from more focused perspectives—such as biological determinants, lipid-composition effects, interface-sensitive spectroscopy, or specific multiscale in silico modeling approaches [30,31,32,33]—the present work adopts a unified, mechanism-oriented framework spanning diverse experimental and computational platforms. Rather than concentrating on specific nanoparticle classes or isolated membrane architectures, this review systematically correlates interaction pathways with key biophysical parameters, including membrane packing density, elasticity, and compositional asymmetry. By explicitly linking mechanistic behavior to the capabilities and limitations of contemporary experimental and simulation techniques, this article provides both a conceptual framework and a practical roadmap for advancing nano–bio interface research and guiding future developments in nanomedicine and biosensing technologies.

## 2. Types of Nanoparticles and Molecular Nanostructures

Nanoparticle systems relevant to membrane interactions can be categorized according to their structural complexity and functional organization rather than purely by chemical composition. The present section therefore emphasizes polymeric, lipid-based, molecular, and hybrid nanostructures that define major design strategies at the bio-interface. While most of these systems are organic or hybrid in character, inorganic nanoparticles such as gold, silver, and zinc oxide—typically surface-modified with organic or polymeric shells—have also been extensively investigated for their interactions with biological membranes, particularly in antimicrobial and toxicological contexts [5,10,14].

### 2.1. Polymeric Nanoparticles: Tunable Platforms for Controlled Delivery

**Figure 2:**
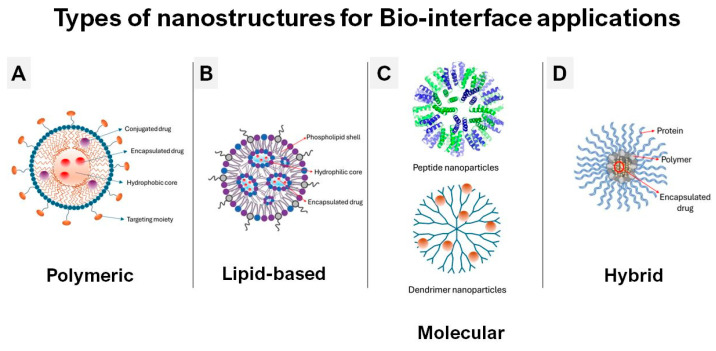
Schematic representation of different types of nanostructures relevant for bio-interface applications. The figure illustrates: (**A**) polymeric nanoparticles, (**B**) lipid-based nanostructures (lipid nanoparticles), (**C**) molecular nanostructures (peptide-based nanoparticles and dendrimers), and (**D**) hybrid nanostructures. Each category exhibits unique structural and functional characteristics that can be tailored to enhance stability, biocompatibility, targeting capability, and controlled drug delivery, supporting their versatile use in biomedical and therapeutic applications (Original schematic).

Polymeric nanoparticles (PNPs) (Figure 2A) are among the most extensively studied nanocarriers due to their tunable physicochemical properties and versatility in encapsulating various therapeutics [34,35]. Key parameters—including size, shape, surface charge, and surface functionalization—strongly influence their biodistribution, cellular uptake, and therapeutic efficacy [36]. Generally, smaller nanoparticles penetrate tissues more efficiently, while spherical morphologies favor internalization compared to rod-like or irregular particles [35,37]. Surface charge modulates interactions with cell membranes and serum proteins: positively charged PNPs exhibit enhanced uptake via electrostatic interactions with negatively charged membranes, whereas surface modifications such as PEGylation or ligand functionalization improve circulation stability and targeting specificity [37,38]. The choice of polymer material is equally critical. Biodegradable polymers provide controlled drug release while degrading into non-toxic byproducts [37,39], whereas biocompatible polymers mitigate immune activation [40]. PNPs can encapsulate both hydrophilic and hydrophobic drugs, shield sensitive molecules from premature degradation, and be engineered for stimuli-responsive release, adding precision to therapeutic delivery [41]. Despite these advantages, translation remains limited by challenges in large-scale production, regulatory standardization, and long-term safety evaluation [34,38]. Overcoming these barriers will require cross-disciplinary advances in polymer chemistry, nanofabrication, and clinical testing.

### 2.2. Lipid-Based Nanostructures: Biocompatibility, Stability, and Clinical Hurdles

Lipid-based nanocarriers, including liposomes and solid lipid nanoparticles (SLNs) (Figure 2B), are inherently biocompatible and biodegradable, making them attractive platforms for drug delivery [42,43]. Their interactions with cell membranes are governed by particle size, surface charge, and lipid composition, parameters that can be tuned to optimize uptake and biodistribution [44]. Incorporation typically proceeds via endocytic pathways, with lipid tailoring (e.g., incorporation of oleic acid) enabling enhanced uptake without altering the underlying mechanism [44]. Liposomes provide flexible encapsulation of hydrophilic and hydrophobic drugs, while SLNs offer additional benefits of drug stabilization and protection from enzymatic degradation [45,46]. However, their stability in physiological conditions remains a central challenge: aggregation, leakage, and rapid clearance can compromise performance [44]. Maintaining colloidal integrity and reproducibility during scale-up are therefore critical hurdles for clinical translation [47]. Although lipid nanocarriers show promise in improving drug bioavailability and reducing systemic toxicity, their interactions with membranes may also trigger inadvertent effects, such as membrane destabilization or immune activation [43,48]. Continued mechanistic studies are essential to balance efficacy with safety.

### 2.3. Peptide Nanostructures and Dendrimers: Molecular Precision at Bio-Interfaces

Peptide nanostructures and dendrimers represent molecularly precise platforms with high biocompatibility and flexible design (Figure 2C). Peptide-based nanostructures self-assemble into diverse morphologies, mimicking native biomolecules and enabling bioinstructive interactions with cells [49,50,51]. Their inherent biodegradability, negligible cytotoxicity, and resistance to enzymatic degradation make them especially attractive for drug delivery and antimicrobial applications [49,50,51,52,53]. Stimuli-responsive designs add further control, releasing therapeutic cargo in response to enzymatic or environmental triggers [51]. Dendrimers, by contrast, are monodisperse, highly branched polymers with tunable size and surface functionality [54]. Their architecture supports efficient drug encapsulation, solubility enhancement, and multivalent ligand presentation, facilitating targeted interactions with biological systems [54,55]. Key challenges for both systems include synthetic scalability, functionalization complexity, and regulatory standardization. Nonetheless, their structural precision and capacity for fine-tuned biological engagement highlight their potential in next-generation nanomedicine [56].

### 2.4. Hybrid Nanostructures: Synergistic Designs for Multifunctional Applications

Hybrid nanostructures integrate distinct material classes—polymeric, lipidic, inorganic, or biomolecular—to synergistically enhance stability, biocompatibility, and functionality [57,58] (Figure 2D). By combining complementary properties, hybrids can prolong circulation, reduce clearance, and enable targeted delivery with improved therapeutic indices [59,60,61]. Their modular design allows tailoring of surface chemistry to modulate immune responses and enhance cellular uptake. At the bio-interface, hybrid nanostructures are subject to the same governing principles as single-material systems: size, shape, charge, and composition dictate uptake and biodistribution, while dynamic processes such as protein corona formation further modulate biological identity [30,62,63]. Importantly, molecular forces (electrostatic, hydrophobic, hydrogen bonding) and redox processes can induce structural changes in adsorbed biomolecules, influencing both efficacy and toxicity [5,57]. The translational potential of hybrid systems is evident in regenerative medicine, imaging, and theranostics—where engineered surfaces can guide stem cell differentiation or enable multifunctional therapies [64]. Yet, issues of safety, unintended responses, and regulatory complexity remain significant barriers [5,65].

## 3. Model Membrane Systems

### 3.1. Langmuir Monolayers: Simplistic Platforms for Interfacial Insights

**Figure 3:**
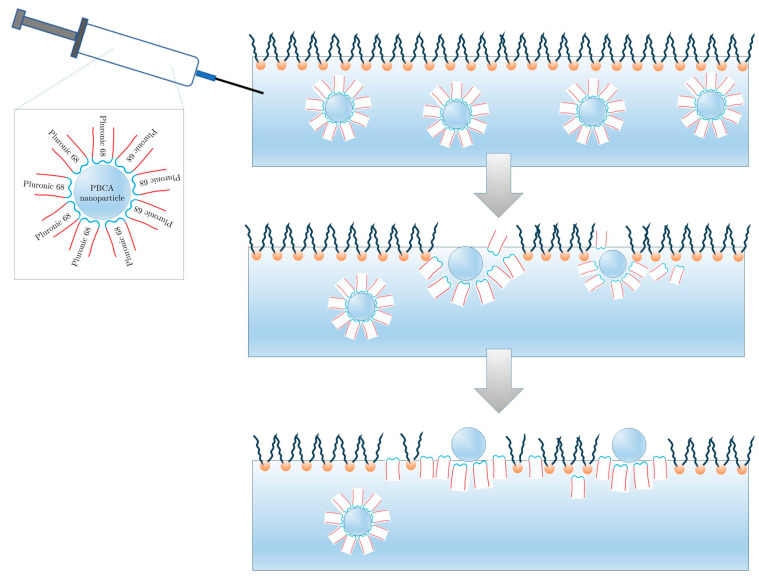
Langmuir monolayer as a model membrane to study the penetration and reorganization of PBCA nanoparticles (with sizes about 200 nm) stabilized by Pluronic 68. Surface area and surface potential changes were monitored under constant pressure. Adapted from Ref. [75] (Yordanov, G. et al., Membranes 2024, 14), licensed under CC BY 4.0; modifications made.

Langmuir monolayers are among the most established model systems for probing membrane structure and dynamics because they capture the two-dimensional character of cellular interfaces under highly controlled conditions [22,66,67]. Composed of amphiphilic molecules organized at the air–water interface, they allow systematic investigation of lipid packing, phase transitions, and molecular interactions with exogenous agents such as drugs, surfactants, and environmental contaminants [67,68,69,70,71]. Compression–isotherm measurements yield thermodynamic parameters that link molecular packing to phase behavior, making monolayers particularly powerful for studying how chemical or nanomaterial perturbations influence membrane mechanics [70,71,72]. Their versatility lies in tunable composition and compatibility with advanced techniques such as X-ray and neutron reflectometry, infrared and sum-frequency generation spectroscopy, and surface potential measurements [72,73,74]. Recent work has extended their utility to nanoparticle studies—for example, PBCA nanoparticles stabilized with Pluronic 68 with sizes about 200 nm were shown to reorganize phospholipid monolayers under constant pressure, providing mechanistic insight into nanoparticle–membrane penetration [75] (Figure 3).

Monolayers can also be transferred to solid supports for combined analyses by convenient microscopy or spectroscopy methods [70]. Despite these advantages, Langmuir monolayers remain simplified models: they lack bilayer architecture, proteins, and the three-dimensional complexity of living membranes [76,77]. While invaluable for reductionist insights, they are best used in combination with complementary systems such as SUVs or vesicles to approximate native biology more closely [78].

### 3.2. SLBs: Stable Two-Dimensional Mimics of Cell Membranes

**Figure 4:**
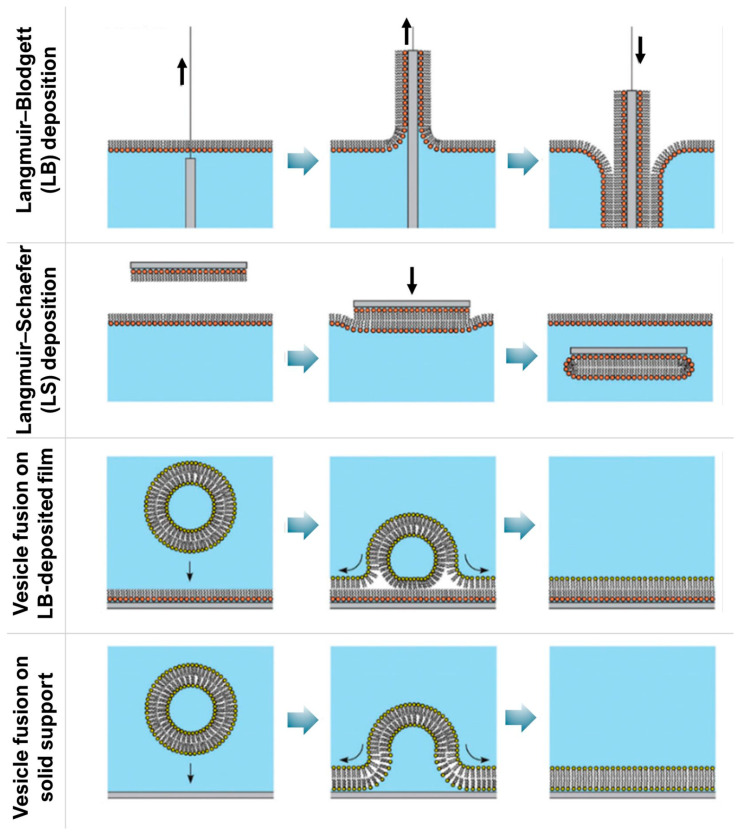
Common preparation methods for SLBs: Langmuir–Blodgett, Langmuir–Schaefer, and vesicle fusion. These approaches enable controlled studies of biomimetic membranes and nanoparticle interactions. Adapted from Ref. [79] (Sondhi et al., Coatings 2020, 10, 981), licensed under CC BY 4.0; modifications made.

SLBs overcome many limitations of monolayers by providing stable, hydrated bilayer structures on solid substrates [79,80]. They are well suited for studying adsorption, binding, and penetration of nanoparticles and biomolecules under physiologically relevant conditions, while remaining accessible to surface-sensitive techniques [79,80,81]. As shown in Figure 4, fabrication strategies such as Langmuir–Blodgett/Schaefer transfer and vesicle fusion [79] provide compositional flexibility, allowing for the incorporation of biologically relevant lipids, notably cholesterol [82].

**Figure 5:**
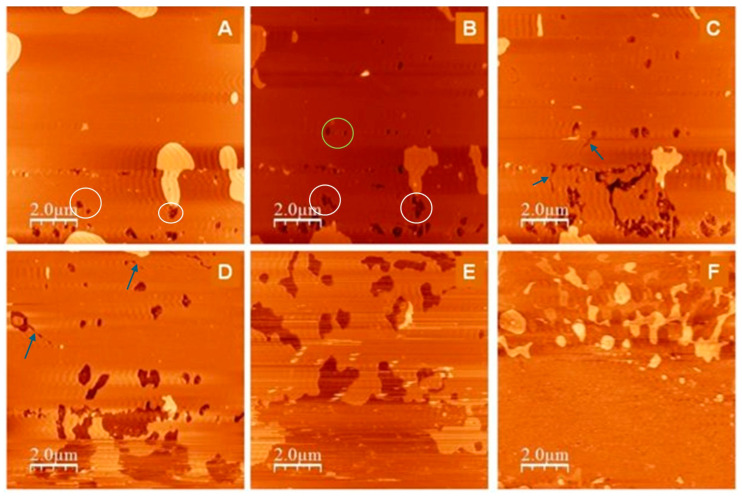
Time-course AFM images showing the PLA_2_-mediated hydrolysis of supported DPPC bilayers. Panels (**A**–**F**) depict the sequential morphological evolution of the bilayer surface after enzyme injection. Brighter regions correspond to intact phospholipid domains, while the most intense bright features likely represent phospholipid multilayers or reorganized and residual bilayer structures formed as intermediate products of enzymatic hydrolysis. Darker regions indicate areas where the bilayer has been hydrolyzed and partially removed. In panels (**A**,**B**), the *white circles* highlight pre-existing double-layer defects that expand over time, illustrating their enlargement during the hydrolytic process. The *green circle* in panel (**B**) marks a newly formed defect attributed to PLA_2_ activity. In panels (**C**,**D**), *blue arrows* indicate the emergence of narrow channel-like features consistent with localized enzymatic penetration and early-stage hydrolysis pathways. The progressive increase in the number and size of dark regions and the appearance of nanoscale channels reflect the time-dependent degradation and reorganization of the DPPC bilayer under PLA_2_-catalyzed hydrolysis [86].

SLBs reveal how nanoparticle surface charge, hydrophobicity, and ion content govern interfacial behavior. For instance, cationic gold nanoparticles preferentially associate with liquid-ordered domains, while nanoparticles can also drive structural rearrangements such as lipid sheath formation [83,84,85]. At the micrometer scale, AFM resolves the structural reorganization of the phospholipid bilayer during PLA_2_ hydrolysis. Although individual enzymatic events are not directly imaged, higher-resolution scans of supported lipid bilayers (SLBs) reveal local depressions and narrow channel-like features—comparable in size to single enzyme molecules—that are consistent with localized hydrolytic activity [86]. As shown in Figure 5, PLA_2_ activity induces progressive disruption of the supported DPPC bilayer, manifested as the growth of darker regions corresponding to hydrolyzed areas.

The sequential AFM images (Figure 5A–F) show the expansion of pre-existing double-layer defects (white circles), formation of new enzymatic defects (green circle), and appearance of channel-like structures (blue arrows) that mark localized hydrolysis and membrane reorganization. These nanoscale depressions and channel-like structures likely represent initial hydrolysis sites and pathways formed by enzymatic penetration and lipid removal, providing indirect morphological evidence of PLA_2_ action on the bilayer surface. Complementary techniques such as QCM-D, SEIRAS, and grazing incidence X-ray or neutron scattering extend the analysis to dynamic and molecular-scale phenomena [82,87]. Yet, SLBs remain oversimplified relative to cellular membranes, lacking embedded proteins, lipid asymmetry, and full compositional diversity. They provide robust mechanistic data but must be integrated with higher-order models for comprehensive understanding [80].

### 3.3. Liposomes: Vesicular Models of Membrane Curvature and Dynamics

**Figure 6:**
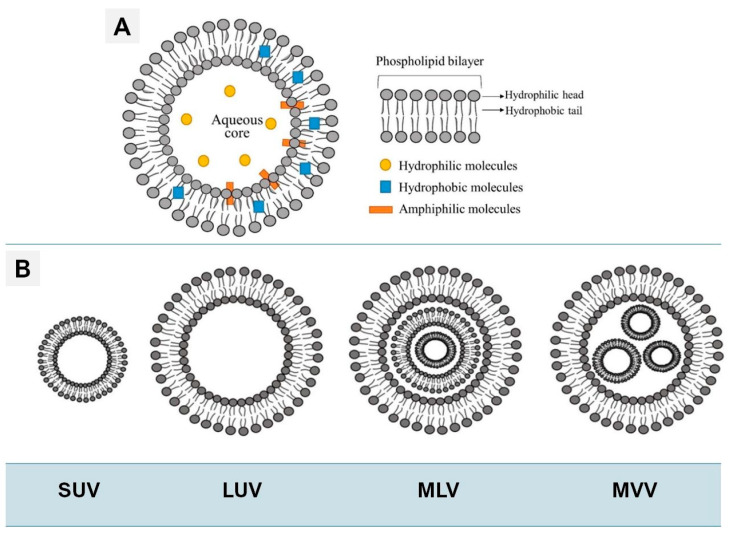
Structure and classification of liposomes. (**A**) Phospholipid bilayer enclosing an aqueous core, with potential incorporation of hydrophilic, hydrophobic, and amphiphilic molecules. (**B**) Liposome types by size and lamellarity: SUVs, large unilamellar vesicles (LUVs), multilamellar vesicles (MLVs), and multivesicular vesicles (MVVs). Adapted from Ref. [79] (Sondhi et al., Coatings 2020, 10, 981), licensed under CC BY 4.0; modifications made.

Liposomes are closed phospholipid bilayers that encapsulate an aqueous core, functioning as versatile three-dimensional membrane models that bridge planar systems with cellular architectures [88,89,90]. Their amphiphilic organization enables the simultaneous encapsulation of hydrophilic compounds within the internal aqueous compartment and hydrophobic molecules within the bilayer matrix, providing a tunable environment for both fundamental and applied investigations. As illustrated in Figure 6 [79], the fundamental liposomal structure consists of a phospholipid bilayer enclosing an aqueous core, a unique architecture that permits the simultaneous encapsulation of hydrophilic, hydrophobic, and amphiphilic cargo (Figure 6A). Depending on the fabrication method, these vesicles are categorized by size and lamellarity into distinct classes (Figure 6B), ranging from small and large unilamellar vesicles (SUVs and LUVs) to more complex multilamellar (MLVs) and multivesicular (MVVs) systems. Owing to their compositional versatility, controllable size, and inherent biocompatibility, liposomes are used as robust platforms for probing membrane permeability, fusion, curvature-induced stress, and dynamic lipid–protein interactions under physiologically relevant conditions [89,90].

Importantly, liposomes can be systematically engineered by modifying lipid composition, surface charge, cholesterol content, and membrane fluidity, enabling the reproduction of diverse membrane phenotypes—from disordered liquid-crystalline phases to tightly packed, raft-like domains. This tunability supports mechanistic studies that investigate how molecular architecture, packing constraints, and environmental factors shape membrane behavior and nanoparticle interactions.

At the translational level, their structural resemblance to biological membranes underpins their widespread use as nanocarriers in drug delivery, where they enhance solubility, stabilize labile compounds, and enable controlled or stimuli-responsive release [88]. Recent advancements have further expanded their applications toward targeted delivery, theranostics, and immunomodulation, solidifying liposomes as both experimental models and clinically relevant nanostructures. Their dual role—as simplified biomimetic analogs and as therapeutic vehicles—establishes them as a critical link between membrane biophysics and nanomedicine.

**Figure 7:**
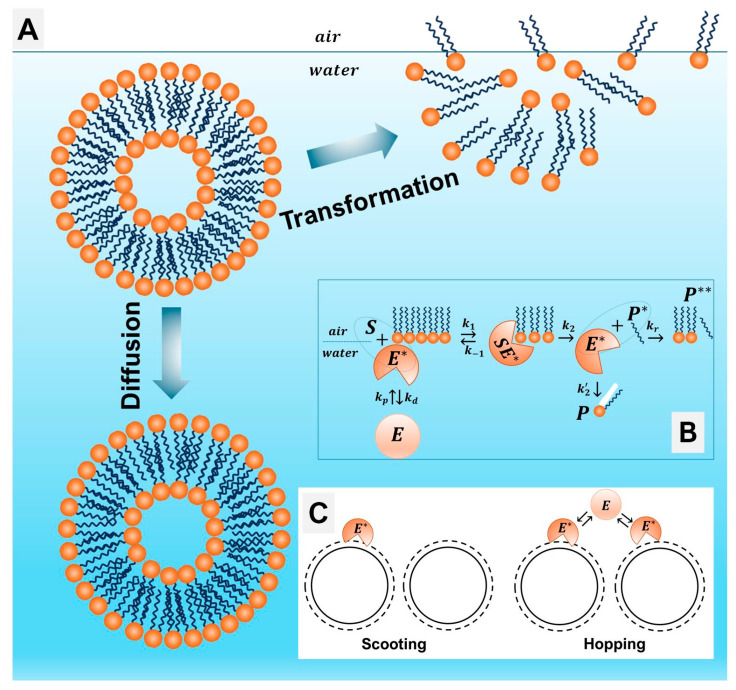
Kinetic models of liposome interfacial catalysis. (**A**) Spreading of liposomes at the air–water interface. (**B**) Michaelis–Menten–type scheme for interfacial enzymatic hydrolysis. The symbols P* and P** denote the two enzymatic reaction products (e.g., fatty acid and lysophospholipid, respectively). Depending on acyl chain length, these products may remain at the interface (longer chains) or dissolve into the aqueous subphase (shorter chains). (**C**) Schematic comparison of ‘scooting’ and ‘hopping’ catalytic modes of PLA_2_ (cf. [95]) (Original schematic).

GUVs enable direct optical visualization of membrane morphology, phase separation, and nanoparticle interactions, including adhesion, pore formation, or lysis [23,91,92]. Their size also permits incorporation of transmembrane proteins, rendering them highly relevant for artificial cell models [93]. SUVs, by on the other hand, offer simplified platforms for kinetic studies [94]. In seminal PLA_2_ experiments has been established interfacial catalysis mechanism that distinguished scooting versus hopping modes of enzyme action [95] (Figure 7).

Liposomes thus provide unique access to curvature-dependent processes and dynamic phenomena not captured by planar models. However, they cannot fully replicate the complexity of living membranes, and their stability during manipulation can be a limiting factor. As with other models, they are most powerful when used complementarily with SLBs and monolayers.

### 3.4. Hybrid and Complex Membrane Mimics: Bridging Reductionist and Native Systems

Hybrid membrane mimics integrate lipid, polymeric, inorganic, and biological components to approximate the structural and functional complexity of native membranes [96,97]. Their composite design enhances stability, adaptability, and functionalization capacity, enabling applications ranging from targeted delivery and biosensing to tissue engineering [98,99,100]. Unique systems such as cell-membrane-coated particles preserve natural biocompatibility and immune evasion [101], while hybrid bilayer membranes (HBMs) incorporate self-assembled monolayers on gold or other substrates to combine robust anchoring with tunable lipid interfaces. Such systems allow incorporation of proteins, peptides, or catalytic motifs, offering closer emulation of selective permeability and dynamic responses [98,102]. This structural complexity of all these nanostructures enables a closer approximation of the native environment found in biological systems, offering new insights into membrane-associated phenomena that are difficult to capture with planar or overly simplified models [78,83,97]. Nevertheless, their complexity introduces fabrication, reproducibility, and regulatory challenges. Achieving consistent biofunctionalization and ensuring safety remain major barriers to clinical translation [98,101]. Even so, hybrid systems represent a critical step toward bridging the gap between simplified models and the dynamic complexity of living membranes.

Collectively, these model membrane systems provide complementary perspectives on nanoparticle–membrane interactions, ranging from the reductionist control of Langmuir monolayers to the three-dimensional dynamics of liposomes and the structural sophistication of hybrid mimics. Each system offers distinct advantages but also inherent limitations, underscoring the necessity of integrating multiple approaches to capture the complexity of biological membranes. When combined with advanced analytical and computational methods, these models form a powerful toolkit for unraveling interfacial mechanisms and guiding the rational design of nanomedicines, biosensors, and diagnostic platforms.

### 3.5. Summary of Model Membrane Systems

Collectively, these model membrane systems—ranging from Langmuir monolayers to supported lipid bilayers (SLBs), liposomes, and hybrid biomimetic constructs—provide complementary perspectives on nanoparticle–membrane interactions. Langmuir monolayers offer reductionist control over interfacial thermodynamics and packing; SLBs extend this insight to hydrated bilayer organization accessible to high-resolution and surface-sensitive methods; liposomes capture curvature and dynamic remodeling; and hybrid systems approach the compositional and functional complexity of native membranes. Each model presents distinct advantages and intrinsic limitations, underscoring the importance of integrating multiple approaches to reproduce the full behavior of biological membranes. When combined with advanced analytical and computational methods, these systems form a hierarchical and versatile toolkit for elucidating interfacial mechanisms and for guiding the rational design of nanomedicines, biosensors, and diagnostic platforms.

## 4. Advanced Model Membrane Systems

Model membrane systems have evolved beyond classical monolayers and supported lipid bilayers (SLBs) toward architectures that more closely approximate the structural complexity and functional heterogeneity of biological membranes. These innovations seek to bridge the gap between simplified systems and native membranes by incorporating compositional asymmetry, multicomponent lipid mixtures, and protein-mediated organization [16,17].

A key development is the fabrication of asymmetric bilayers, which mimic leaflet-specific lipid distributions found in living cells. These systems allow investigation of how trans-bilayer asymmetry modulates adsorption, insertion, and membrane destabilization, thereby improving physiological relevance [24]. Complementing synthetic models, membrane-derived vesicles preserve native lipid–protein heterogeneity, including microdomains and raft-like structures, enabling studies of receptor-mediated interactions and nanoscale membrane remodeling that are difficult to reproduce in minimal systems [23].

Increasing attention has also been directed toward advanced planar membrane platforms that offer improved structural control, enhanced stability, and compatibility with high-resolution analytical tools. Recent progress includes hybrid polymer–lipid interfaces, functionalized supports, and engineered bilayers that integrate transport proteins or receptors, broadening the scope of mechanistic studies [20,27].

Collectively, these advanced membrane platforms extend the capabilities of traditional systems by capturing additional structural and dynamic features of biological membranes. Their complementary use alongside classical models provides multiscale insight into membrane organization and supports the mechanistic study of nano–bio interactions under conditions that better reflect physiological complexity [16,17,24,27].

## 5. Experimental Methods: Multiscale Approaches to the Nano–Bio Interface

A variety of experimental and computational approaches have been developed to probe nanoparticle (NP)–membrane interactions, each offering complementary perspectives on structure, molecular mechanics, and dynamics. Key methods include Langmuir trough measurements, Brewster Angle Microscopy (BAM), atomic force microscopy (AFM), quartz crystal microbalance with dissipation monitoring (QCM-D), fluorescence microscopy and spectroscopy, neutron and X-ray reflectometry, and molecular dynamics (MD) simulations.

Together, these tools provide a multiscale view of interfacial events, from thermodynamic signatures and nanoscale morphology to atomistic mechanisms. Their integration has revealed how NP size, charge, and surface chemistry govern adsorption, penetration, and disruption, while also highlighting challenges in reproducibility and biological relevance. The following subsections outline the most widely used methods and their specific contributions.

### 5.1. Langmuir Trough—Thermodynamic Signatures of Interfacial Events

**Figure 8:**
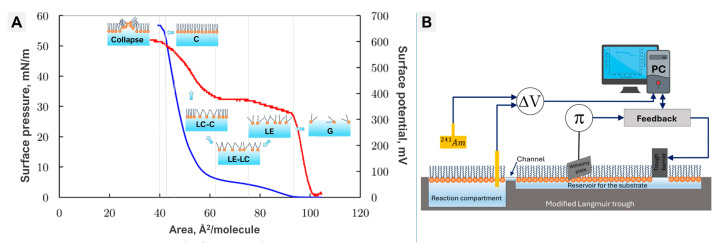
Langmuir trough configurations and complementary isotherms. (**A**) Compression of a DPPC monolayer at the air–water interface showing surface pressure vs. area (π–A, blue) and surface potential vs. area (ΔV–A, red). As the monolayer is compressed, it passes through the characteristic states of saturated phospholipids: a gaseous (G) region at large molecular areas; a liquid-expanded (LE) region with disordered chains; and a liquid-expanded/liquid-condensed (LE–LC) coexistence region representing the first-order transition between expanded and ordered molecular arrangements. A pure liquid-condensed phase does not occur immediately after this transition; instead, the monolayer gradually enters a liquid-condensed/condensed (LC–C) region, where ordered domains continuously increase in density until the film becomes condensed-phase–rich. Once condensed-phase enrichment is reached, the surface potential no longer changes substantially, and ΔV attains its plateau value (POTU) at a molecular area of approximately 45 Å^2^·molecule^−1^. Further compression produces a fully condensed (C) state with minimal compressibility, and continued compression beyond this state results in collapse, where the monolayer buckles into three-dimensional structures. Because π–A senses changes in lateral packing while ΔV–A responds to molecular dipole orientation, the ΔV–A curve typically begins rising at larger molecular areas (e.g., ~100 Å^2^·molecule^−1^), whereas the initial increase in surface pressure occurs at smaller areas (e.g., ~90 Å^2^·molecule^−1^). Insets illustrate representative molecular arrangements in each region. (**B**) Schematic of a modified “zero-order” Langmuir trough incorporating a reaction compartment, a substrate reservoir, and a feedback-controlled barrier system to maintain constant surface pressure. A ^241^Am surface-potential probe may be integrated to allow simultaneous ΔV measurements during monolayer compression. Panel B adapted from Yordanov G. et al. [75], Membranes 2024, 14, CC BY 4.0, with modifications.

The Langmuir trough remains a cornerstone technique for investigating NP–membrane interactions at lipid monolayers, offering precise control over molecular area and high-resolution surface pressure–area (π–A) isotherms [103,104,105]. By systematically compressing amphiphilic films, the Langmuir trough enables identification of distinct phase transitions—gaseous (G), liquid-expanded (LE), and the liquid-expanded/liquid-condensed (LE–LC) coexistence region associated with the first-order transition in saturated phospholipids—as well as the subsequent liquid-condensed/condensed (LC–C) region, where ordered domains densify progressively until the monolayer becomes condensed-phase–rich. Notably, a pure liquid-condensed phase does not appear immediately after the LE–LC transition; instead, the monolayer evolves directly toward the condensed state under continued compression. The π–A and ΔV–A isotherms (Figure 8A) provide complementary thermodynamic descriptions of the monolayer state. Surface pressure (π) reflects the lateral equation of state and increases as molecules pack from G to LE and into the LC–C region, whereas surface potential (ΔV) reports changes in the net interfacial dipole moment associated with headgroup orientation, hydration, and counter-ion rearrangements. Consequently, ΔV often anticipates π during early compression—for example, in the data shown in Figure 8A, the first ΔV change appears near ~100 Å^2^·molecule^−1^, while the initial π rise occurs near ~90 Å^2^·molecule^−1^. As the monolayer becomes condensed-phase–rich, ΔV no longer increases appreciably and reaches a characteristic plateau (POTU), reflecting maximally ordered dipole orientation, typically around ~45 Å^2^·molecule^−1^ for DPPC. Together, these thermodynamic quantities define the G → LE → LE–LC → LC–C → C sequence of monolayer organization and its approach to collapse under controlled compression. Such monolayer measurements provide direct insight into nanoparticle adsorption, insertion, disruption, and lipid reorganization [73,103,104]. Advanced implementations—including “zero-order” trough designs equipped with feedback-controlled barriers and surface-potential probes—enable dynamic studies of enzymatic reactions such as phospholipase-mediated hydrolysis (Figure 8B) [22,75].

The method is highly sensitive to NP physicochemical parameters: size, charge density, surface coatings, and anisotropy all modulate insertion pressure, adsorption depth, and monolayer perturbation [75,105,106]. The robustness of π–A measurements is strengthened by integration with complementary methods. AFM, fluorescence microscopy, and neutron reflectivity validate molecular-scale interpretations [107,108,109,110], while synchrotron-based reflectivity and temperature-controlled troughs enhance precision under complex conditions [106,111]. Together, these combinations provide mechanistic detail not accessible to single methods alone. Despite its versatility, the trough is limited by the simplified nature of monolayers and by assumptions of classical thermodynamics, which can restrict accuracy in multicomponent or biologically complex systems [73,88,112]. Reproducibility is high under well-defined conditions, but decreases when applied to vesicular or protein-containing systems due to environmental variability [113,114].

In essence, Langmuir trough experiments offer exceptional sensitivity and precision for probing nanoparticle–lipid interactions at the air–water interface. Their enduring relevance lies in their adaptability and compatibility with complementary analytical techniques, though advancing toward realistic biological mimicry will require integration with multidimensional and time-resolved methods [113,114,115,116].

### 5.2. Brewster Angle Microscopy (BAM)—Real-Time Morphology of Monolayers

**Figure 9:**
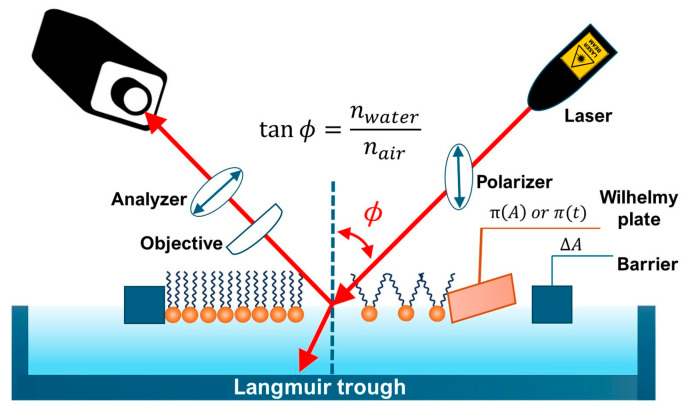
Brewster Angle Microscopy (BAM). *Principle:* at the Brewster angle, p-polarized light is not reflected by pure water; monolayers alter the interfacial refractive index to generate contrast (cf. [118]) (Original schematic).

Brewster Angle Microscopy (BAM) is indispensable for visualizing the morphology and phase behavior of lipid monolayers at the air–water interface in a label-free, noninvasive manner [117,118]. By exploiting the absence of reflected p-polarized light at the Brewster angle, BAM reveals contrast when a monolayer alters the refractive index, enabling direct visualization of domain structures, film heterogeneity, and molecular ordering (Figure 9) [118,119,120].

The method excels at capturing dynamic processes such as domain nucleation, growth, and collapse, providing real-time insight into phase transitions and molecular reorganization [121]. Its sensitivity to optical anisotropy allows qualitative and semi-quantitative mapping of film thickness and molecular orientation, which can be further refined through calibration and autocorrelation analysis [122,123,124,125,126]. BAM has proven especially valuable for monitoring how nanoparticles adsorb, penetrate, or reorganize lipid monolayers, linking mesoscale morphological changes to interfacial mechanisms [127,128].

When combined with Langmuir trough data, BAM adds morphological context to surface pressure isotherms, enhancing interpretation of lipid packing and phase behavior [129,130]. Despite its lower spatial resolution compared with AFM and limited chemical specificity without auxiliary probes, BAM remains unmatched for real-time, mesoscale imaging of monolayers. Overall, it provides a powerful optical window into the mesoscale dynamics of interfacial systems. Its integration with complementary methods such as IRRAS, X-ray scattering, or fluorescence microscopy further strengthens its role as a bridge between thermodynamic measurements and molecular-level characterization.

### 5.3. Atomic Force Microscopy (AFM)—Surface Topography of SLBs

**Figure 10:**
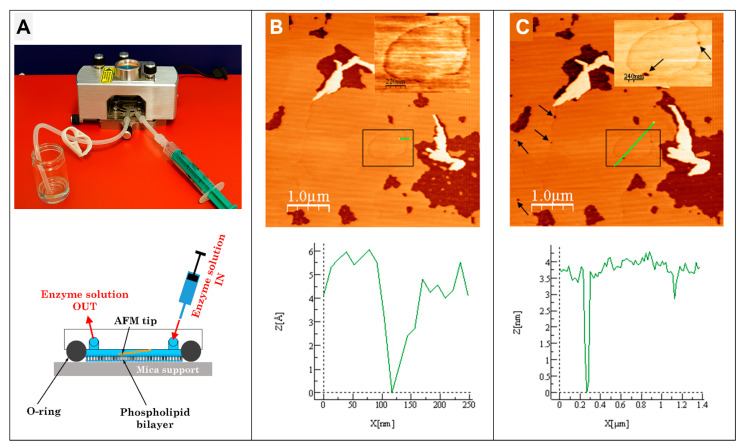
Atomic Force Microscopy (AFM) applied to supported lipid bilayers. (**A**) Liquid-cell setup for imaging supported lipid bilayers (SLBs) under native conditions. (**Upper image**): Photograph of the Nanoscope V AFM optical head equipped with a mounted liquid cell connected to a syringe for enzyme injection. (**Lower image**): Schematic representation of the AFM liquid-cell configuration adapted for lipolysis experiments on supported phospholipid bilayers. (**B**) AFM height image recorded 30 min after injection of the PLA_2_-active Vipoxin toxin. A partially formed supported lipid bilayer is observed, in which several pre-existing dark patches correspond to areas of incomplete SLB coverage. These regions—referred to in the literature as *structural bilayer defects*—either remain unchanged under the action of PLA_2_ or grow only very slowly, in contrast to *compositional defects* generated by individual enzyme molecules. The latter appear on the nanoscale as shallow depressions (0.3–0.5 nm deep). A magnified view of the boxed region highlights their characteristic morphology [86]. The green line indicates the position of the height cross-section shown below. (**C**) AFM height image recorded 37 min after enzyme injection. Additional nanoscale and small microscale hydrolytic defects (indicated by arrows) have appeared, reflecting their lateral expansion and the merging of neighboring hydrolysis sites into larger continuous defects. The corresponding height profile along the green line is shown below. Image size: 4.5 μm × 4.5 μm.

Atomic Force Microscopy (AFM) provides nanometer-scale resolution for studying supported lipid bilayers (SLBs) and other membrane models under fully hydrated conditions [131,132]. When operated with a liquid cell, AFM preserves the native state of biological samples, enabling direct visualization of bilayer architecture, membrane remodeling, and nanoparticle interactions in real time [86]. Figure 10 illustrates the application of this technique for real-time observation of phospholipid bilayers under enzymatic action. The liquid-cell configuration (Figure 10A) allows imaging of supported lipid bilayers in their native aqueous environment while maintaining controlled exchange of the enzyme solution, enabling continuous monitoring of bilayer morphology during lipolysis.

After injection of the PLA_2_-active Vipoxin toxin, the AFM images (Figure 10B) reveal that during the so-called lag phase nanoscale depressions begin to appear, with typical depths of approximately 0.3–0.5 nm. These shallow features correspond to *compositional defects*—local accumulations of hydrolysis products (fatty acids and lysophospholipids) generated by individual enzyme molecules—which remain embedded within the intact bilayer structure during the lag phase. As hydrolysis progresses (Figure 10C), these nanoscale sites expand laterally and may merge with neighboring defects, giving rise first to small microscale defects and later to larger bilayer discontinuities that reflect the advancing stages of lipid degradation. In contrast, the larger pre-existing dark patches visible in the images (Figure 10B) represent *structural bilayer defects* arising from incomplete SLB formation and remain largely unchanged under enzymatic action [86]. This demonstrates the capability of AFM to resolve both the earliest nanoscale manifestations of PLA_2_ activity and the subsequent mesoscale evolution of bilayer disruption.

The technique provides detailed surface topography while force spectroscopy modes quantify biomolecular interactions at the single-molecule level, revealing binding strengths, stability, and specificity [132,133]. AFM has been successfully employed in elucidating how lipid composition, phase state, and lateral heterogeneity influence membrane structure and function [131], as well as in revealing the mechanisms of action of membrane-active agents such as antimicrobial peptides [134,135]. High-resolution AFM imaging has captured nanoscale details of SLB morphology and associated biomolecules, allowing the observation of dynamic processes including protein aggregation and membrane remodeling [80,136]. Furthermore, AFM-based molecular recognition force spectroscopy (MRFS) has quantified the interaction forces between individual biomolecules and lipid membranes [132,133], providing valuable insights into the strength, stability, and specificity of these interactions [135]. The main advantage of AFM lies in combining structural imaging with mechanistic data, including kinetics extracted from sequential imaging of enzymatic or nanoparticle processes. Limitations include the need for defect-free bilayers and careful interpretation, as simplified SLBs cannot fully recapitulate the complexity of native membranes. Nevertheless, the AFM as an experimental method remains a core laboratory technique of membrane biophysics as it uniquely links nanoscale structure and mechanics, providing indispensable mechanistic insights into NP–membrane interactions. Future progress will rely on high-speed AFM and multimodal integration to capture fast dynamics and chemical specificity.

### 5.4. Quartz Crystal Microbalance with Dissipation Monitoring (QCM-D)—Real-Time Adsorption and Viscoelasticity

QCM-D enables real-time monitoring of nanoparticle adsorption, lipid bilayer formation, and viscoelastic changes at membrane interfaces [137,138,139,140,141,142]. By measuring shifts in resonance frequency (Δf) and energy dissipation (ΔD), it distinguishes between rigid mass uptake and structural rearrangements such as bilayer softening, vesicle rupture, or nanoparticle binding. The high sensitivity of QCM-D to mass and viscoelastic changes enables precise evaluation of interactions between nanoparticles and SLBs, ensures accurate assessment of bilayer quality and minimizes experimental artifacts from poorly formed membranes [143]. Frequency shifts reveal mass changes during adsorption or desorption, while dissipation changes provide information on viscoelastic alterations in the membrane structure [142]. By integrating QCM-D with complementary analytical methods, such as field-effect transistors, it is possible to simultaneously monitor mechanical and electrical changes, thereby providing deeper mechanistic understanding of nanoparticle translocation across lipid bilayers [144]. QCM-D can also detect the formation of transient pores or permanent defects in the bilayer—critical indicators of nanoparticle-induced membrane disruption [144]. Integrating QCM-D with complementary methods, such as FRAP or optical reflectometry, enriches analysis of lipid exchange and nanoparticle translocation [145,146]. While QCM-D provides robust kinetic and viscoelastic information, it is inherently limited by the simplified nature of model membranes. Translating findings to heterogeneous, protein-rich cellular membranes requires careful interpretation.

Overall, QCM-D delivers quantitative, real-time insight into nanoparticle–bilayer interactions. Its strength lies in sensitivity to dynamic structural changes, but its greatest impact comes when integrated with orthogonal imaging and spectroscopic approaches.

### 5.5. Fluorescence Microscopy and Spectroscopy—Localization and Functional Impact

**Figure 11:**
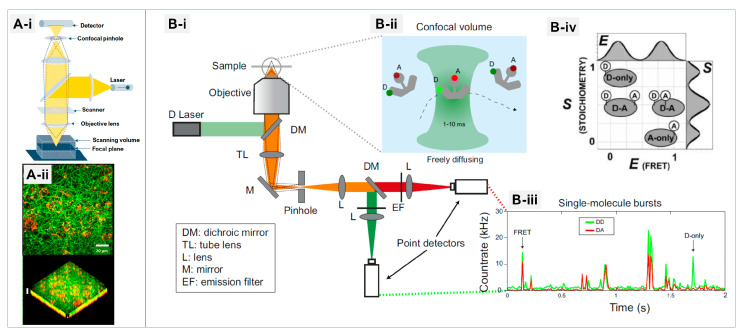
Fluorescence microscopy and spectroscopy. (**A**) Confocal laser scanning microscopy (CLSM): principle (**i**) and Z-projection of fibrin clots on HMEC-1 cells (**ii**). (**B**) Single-molecule FRET: schematic setup (**i**), labeled molecule passage (**ii**), photon bursts (**iii**), and E–S histogram distinguishing populations (**iv**). Adapted from Ref. [147] (Lagali (Ed.), IntechOpen, 2013) CC BY 3.0 (https://creativecommons.org/licenses/by/3.0/ (accessed on 1 March 2026)); changes made.

Fluorescence-based techniques are central to visualizing nanoparticle localization and quantifying membrane perturbations with high sensitivity and chemical specificity [147,148,149]. Figure 11 provides an overview of advanced fluorescence-based characterization techniques, contrasting the imaging capabilities of Confocal Laser Scanning Microscopy (CLSM) with the molecular-level sensitivity of Single-molecule FRET [147]. Confocal laser scanning microscopy (CLSM) enables three-dimensional reconstruction of NP distribution in cells and co-localization with organelles (Figure 11A). By sequentially scanning the laser point-by-point across the sample, CLSM generates a series of optical sections that can be computationally combined to reconstruct a detailed three-dimensional (3D) representation of the specimen (Figure 11(A-i)). As a representative example, Figure 11(A-ii) presents both two-dimensional (2D) and 3D reconstructions showing a Z-projection of a 10 μm-thick region near the cell surface, illustrating fibrin clots anchored to the cell membrane when formed with control plasma. Super-resolution methods such as STED microscopy further resolve NP–organelle and NP–membrane interactions at nanometer precision [150,151].

Fluorescence spectroscopy adds mechanistic depth: Förster resonance energy transfer (FRET) reports on nanoscale membrane rearrangements, while solvatochromic probes map polarity and lipid packing changes induced by NPs (Figure 11B) [149,152]. In this setup, excitation light is directed into the sample through a high numerical aperture objective, and the emitted fluorescence is collected through the same optical path, passed through a confocal pinhole to reject out-of-focus light, and then spectrally separated into donor and acceptor channels. As molecules diffuse through the femtoliter-scale confocal volume (Figure 11(B-ii)), they produce discrete photon bursts (Figure 11(B-iii)), from which FRET efficiencies can be extracted. This allows for the detection of both single- and double-labeled species in real time. By plotting FRET efficiency (E) against stoichiometry (S) in alternating-laser excitation (ALEX) or pulsed interleaved excitation (PIE) experiments, distinct molecular populations—such as donor-only, acceptor-only, and donor–acceptor pairs—can be clearly resolved (Figure 11(B-iv)), enabling precise characterization of molecular heterogeneity at the single-molecule level. Single-molecule FRET (smFRET), on the other hand, captures molecular heterogeneity, providing insights into transient events otherwise obscured in ensemble averages.

Challenges include dye photobleaching and reliance on labeling, but integration with Raman spectroscopy or label-free probes mitigates these limitations and enhances chemical specificity [148,153]. As a whole, fluorescence methods provide unparalleled sensitivity and versatility for tracking NP localization and membrane remodeling. Their continued integration with complementary label-free and single-molecule techniques promises deeper mechanistic understanding of nano–bio interactions.

### 5.6. Neutron and X-Ray Reflectometry—Structural Profiling Across the Membrane Interface

**Figure 12:**
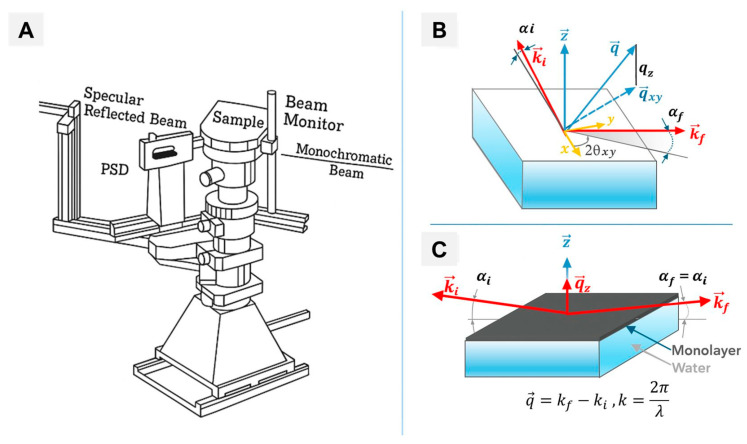
X-ray scattering at the air–liquid interface. (**A**) BW1 liquid surface diffractometer at HASYLAB/DESY used for synchrotron X-ray measurements. (**B**) Geometry for grazing-incidence X-ray diffraction (GIXD). (**C**) Geometry for specular X-ray reflectivity (XR) (Original schematic).

Neutron reflectometry (NR) and X-ray reflectometry (XRR) provide sub-nanometer resolution of membrane architecture and nanoparticle interactions [154,155,156]. Grazing-incidence X-ray diffraction (GIXD) and specular XRR resolve lateral and vertical organization of Langmuir monolayers (Figure 12) [86,155,156,157,158].

X-ray scattering experiments, performed with high sensitivity using liquid surface diffractometers equipped with advanced electronic modules for precise control of thermodynamic parameters such as surface pressure and temperature, allow the investigation of phase transitions and interfacial reactions (Figure 12A) [86,158]. Diffraction and reflection of X-rays from monolayers at the air/water interface are special cases of surface scattering. Langmuir monolayers, often comprising coexisting phases with distinct angular orientations, can be considered as the two-dimensional equivalent of a three-dimensional powder. Consequently, the principles of grazing-incidence X-ray scattering theory are adapted from classical 3D crystallography for two specific geometries (Figure 12B). One geometry is applied to probe the lateral structure of the monolayer via GIXD (Figure 12B), while the other is used to extract information about the vertical structure of a monomolecular layer through specular XR measurements (Figure 12C).

As an experimental counterpart, NR can precisely determine the separation distance between nanoparticles and SLBs, providing critical insights into molecular recognition processes and nanoparticle–membrane interactions [159]. Moreover, NR’s sensitivity to light elements and its ability to probe samples under physiological conditions make it particularly suitable for biological systems [160,161]. XRR, in turn, provides complementary structural insights into soft matter systems, including lipid membranes and their interactions with nanoparticles, benefiting from continuous advancements in instrumentation and computational analysis [162]. When combined, NR and XRR overcome individual methodological limitations—such as neutron contrast constraints—yielding a more complete structural and dynamic picture of membrane-associated phenomena [161]. These techniques are uniquely suited to quantify nanoparticle adsorption depth, membrane thinning, and lipid reorganization, including curvature-dependent processes such as lipid sheathing of nanoparticles [159]. Advances in synchrotron X-rays and neutron sources have also expanded temporal resolution and sensitivity [86,158,162].

Despite the need for complex modeling and complementary validation, combined NR and XRR overcome individual limitations, providing a uniquely complete structural profile of NP–membrane interactions. These techniques deliver unmatched quantitative structural resolution. Their integration with AFM, QCM-D, or fluorescence imaging transforms them from purely structural probes into mechanistic tools for nanomedicine and biosensing applications.

### 5.7. Molecular Dynamics (MD) Simulations—Mechanistic Insight at the Molecular Level

Computational modeling has become essential for elucidating membrane-mediated processes and nanoparticle behavior at biological interfaces, offering mechanistic insights that complement experimental observations [6,7,26]. In particular, molecular dynamics (MD) simulations—employing both all-atom and coarse-grained representations—enable the direct interrogation of nanoparticle–membrane interactions across multiple spatial and temporal scales [7,26].

All-atom MD simulations provide atomic-level resolution of lipid–nanoparticle interactions, allowing for the detailed characterization of permeation pathways, ligand insertion, local lipid rearrangements, and configurational transitions often inaccessible to experimental techniques [163,164,165,166]. Complementarily, coarse-grained MD extends simulations to larger membrane patches and longer timescales, facilitating the exploration of collective membrane responses such as lipid reorganization, nanoparticle insertion, lateral domain restructuring, and mesoscale deformation [6,7,26]. This multiscale capability is particularly valuable, as nanoparticle–membrane interactions inherently emerge from the coupling of local molecular events with cooperative, collective membrane behavior.

Beyond structural visualization, MD simulations enable the systematic evaluation of key physicochemical parameters—including nanoparticle size, geometry, surface chemistry, ligand architecture, and hydrophobicity—and their specific influence on membrane organization, energetics, and remodeling [163,164,167,168,169]. However, distinct challenges remain. Computational studies face inherent limitations related to force-field accuracy, sampling efficiency, accessible spatiotemporal scales, and the representation of complex compositional heterogeneity. These constraints underscore the critical need for cross-validation against experimental data and the adoption of integrated experimental–theoretical frameworks [26,170].

**Figure 13:**
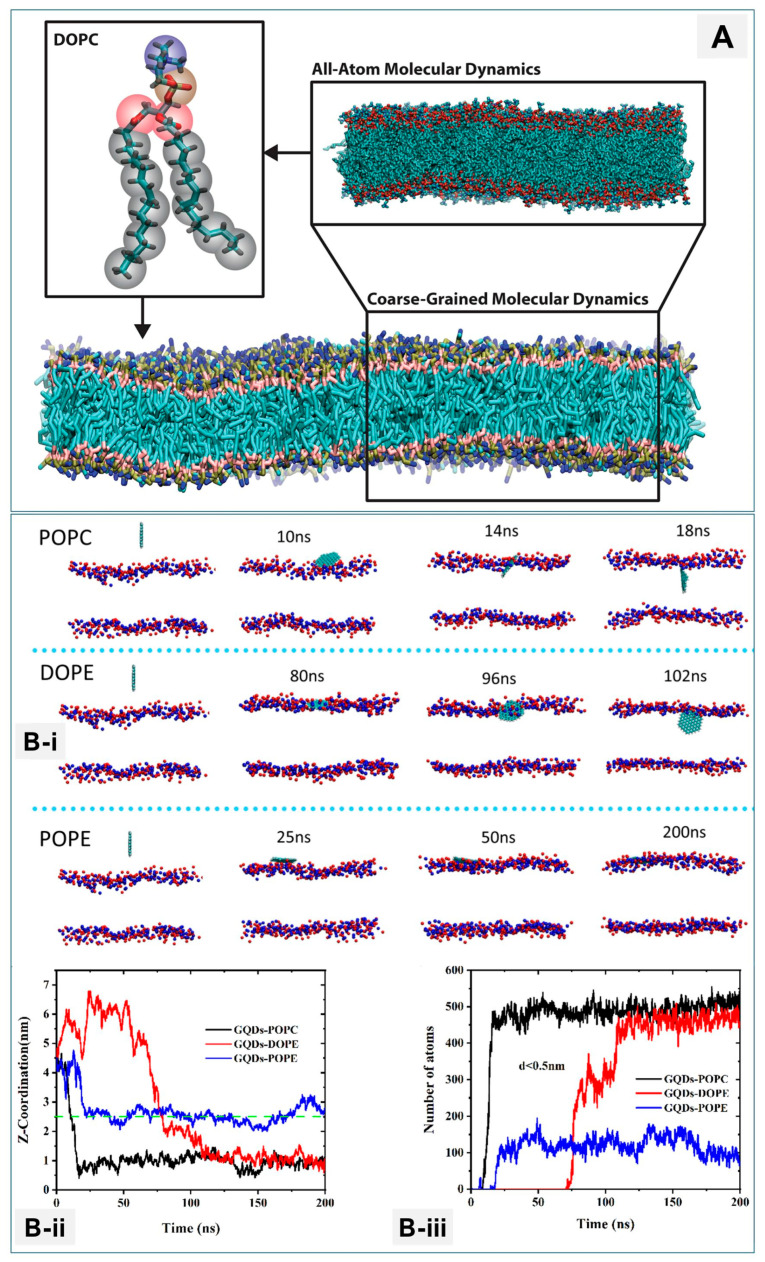
Multiscale molecular dynamics simulations of nanoparticle–membrane interactions. (**A**) Representative all-atom (**upper right**) and coarse-grained Martini (**bottom**) molecular dynamics snapshots of a DOPC/DOPS lipid bilayer, illustrating the contrast between atomic-level and bead-based membrane representations. The coarse-grained mapping of a single DOPC lipid is shown schematically (upper left). Adapted from Ref. [171] under CC BY 4.0; modifications made. (**B**) Molecular dynamics simulations of graphene quantum dot (GQD) interactions with phospholipid bilayers of different packing densities. (**B-i**) Time-resolved snapshots demonstrate GQD penetration into POPC and DOPE bilayers and surface adsorption on densely packed POPE membranes. (**B-ii**) Time evolution of the GQD position along the membrane normal. (**B-iii**) Number of GQD–lipid atomic contacts over time, indicating stronger interfacial association with less densely packed membranes. Adapted from Ref. [166] under CC BY 4.0; modifications made.

Figure 13 illustrates the multiscale role of molecular dynamics simulations in resolving nanoparticle–membrane interactions.

Figure 13A highlights the complementary use of all-atom and coarse-grained representations, demonstrating how atomic-level detail and mesoscale membrane organization can be captured within a unified computational framework [171]. Figure 13B(i–iii) further shows how coarse-grained MD reveals packing-dependent nanoparticle behavior: graphene quantum dots penetrate fluid POPC and DOPE bilayers but remain adsorbed on the more densely packed POPE surface. Time-resolved positional and contact analyses indicate that reduced lipid packing density lowers the energetic barrier for insertion, enabling stable incorporation within the bilayer [166]. Together, these simulations provide molecular-level insight into the structural and energetic determinants governing nanoparticle translocation across lipid membranes.

Temporal and spatial resolution at femtosecond-to-microsecond and Ångström-to-nanometer scales allows detection of transient events such as lipid flip-flops or local deformation [172]. MD complements experiments by providing mechanistic interpretation of fluorescence spectroscopy, reflectometry, and microscopy data [167,173]. Increasingly, MD also serves as a predictive design tool, guiding nanoparticle engineering for drug delivery and imaging applications [170]. However, accuracy depends on force-field fidelity and accessible timescales, which limit modeling of long-term membrane remodeling. Advances in enhanced sampling, coarse-graining, and multiscale integration are helping overcome these barriers [170,173]. In conclusion, MD simulations provide unique mechanistic insight and predictive capacity, extending experimental findings to molecular detail. Continued integration with experimental benchmarks will be critical for their role in rational nanomedicine design.

Collectively, the above experimental and computational approaches provide complementary perspectives on NP–membrane interactions, spanning thermodynamic signatures, mesoscale morphology, nanoscale topography, and atomistic trajectories. Their integrative application enables multiscale analysis of adsorption, disruption, and remodeling processes, bridging the gap between simplified model systems and the dynamic complexity of living membranes. This convergence not only advances fundamental understanding but also informs the rational design of nanomedicines, biosensors, and toxicity assessment platforms.

## 6. Mechanisms of Interaction and Illustrative Case Studies

### 6.1. Mechanisms and Types of Nanoparticles–Membrane Interactions

**Figure 14:**
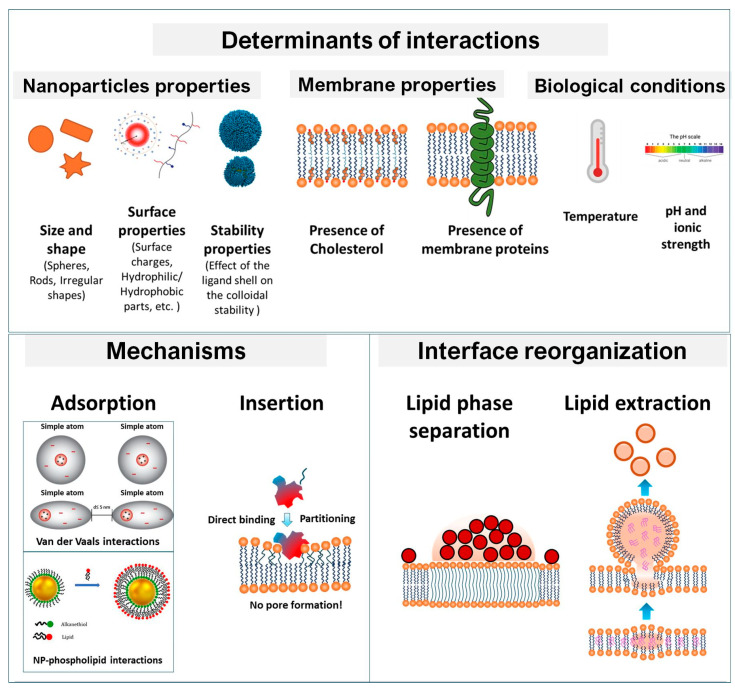
Determinants, mechanisms, and reorganization pathways in nanoparticle–membrane interactions. Key factors include nanoparticle physicochemical properties (size, shape, surface chemistry, hydrophilic/hydrophobic balance, colloidal stability), membrane composition (cholesterol, proteins), and biological conditions (temperature, pH, ionic strength). At the molecular level, these interactions arise from a combination of covalent or coordination bonding (e.g., thiol–gold, silane–oxide), electrostatic attraction or repulsion, hydrogen bonding, hydrophobic partitioning, and van der Waals dispersion forces. Interaction mechanisms involve adsorption via van der Waals forces and NP–phospholipid contacts, or insertion through direct binding or hydrophobic partitioning. Subsequent reorganization may trigger lipid phase separation or extraction, ultimately affecting membrane stability and disruption. (Original schematic).

Nanoparticle–membrane interactions emerge from a finely balanced interplay of physical, chemical, and biological factors, proceeding via distinct pathways such as surface adsorption, bilayer insertion, or combined mechanisms (Figure 14). At the molecular level, these interactions arise from several fundamental force types, including covalent or coordination bonding (e.g., thiol–gold, silane–oxide linkages), electrostatic attraction or repulsion, hydrogen bonding, hydrophobic partitioning, and van der Waals dispersion forces. Adsorption, often the initial stage, is mediated by electrostatic forces, van der Waals interactions, and specific ligand–lipid affinities [174,175]. Insertion involves the penetration of nanoparticles into the hydrophobic core of lipid bilayers, analogous to transmembrane protein incorporation [169,176]. The balance between these mechanisms is governed by nanoparticle surface chemistry (charge, hydrophobicity, ligand architecture), membrane composition and organization (cholesterol, sphingolipids, proteins), and the dynamic state of the membrane, including its fluidity and phase behavior [177,178]. The relative contribution of these forces determines whether nanoparticles remain adsorbed at the surface, partially insert into the headgroup region, or fully penetrate the bilayer core. Surface charge plays a decisive role in early binding events, yet it cannot be considered separately from hydrophobic contributions and ligand conformational flexibility [33,84]. Ligand lipophilicity and structural adaptability influence both adsorption kinetics and membrane perturbation, with intermediate lipophilicity often minimizing energetic barriers to bilayer penetration [168]. Ligand stability further determines the extent of membrane disruption: labile surfactant-type ligands may dissociate and insert into lipid leaflets, reducing bilayer thickness, whereas stable polymeric or polyelectrolyte coatings tend to preserve membrane integrity despite persistent adsorption [179].

Membrane composition critically modulates these interactions. Cholesterol can either enhance or inhibit nanoparticle binding, depending on particle size, surface chemistry, and concentration [180]. Sphingolipids and leaflet asymmetry are also decisive as the loss of asymmetry reduces nanoparticle-induced hemolysis, which was observed for silica nanoparticles in eryptotic versus healthy erythrocytes [181]. Protein components introduce further complexity, with peripheral membrane proteins facilitating or hindering nanoparticle attachment depending on their charge distribution and binding sites [182]. Variations in lipid headgroup tilt, acyl chain saturation, and packing density modulate bilayer resilience, with more saturated chains generally conferring increased resistance [182].

Membrane fluidity and phase behavior add another mechanistic layer. Nanoparticles exhibit phase-selective binding—cationic particles often preferentially adsorb to liquid-disordered regions with lower bending modulus, while hydrophobic nanoparticles may initially insert into disordered domains before relocating to ordered regions [177,183]. Amphiphilic nanoparticles can localize at phase boundaries or suppress lipid phase separation entirely, leading to bilayer-embedded ordered aggregates [178]. Such remodeling underscores the capacity of nanoparticles not only to respond to but also to actively reorganize membrane architecture.

Ultimately, the outcome of nanoparticle–membrane engagement—whether membrane destabilization through pore formation, lipid extraction, and leaflet asymmetry loss, or stabilization via ordered lipid recruitment and curvature matching—depends on the coordinated influence of nanoparticle physicochemical properties, membrane structural features, and the capacity for dynamic reorganization at the bio–nano interface.

To consolidate the diverse interaction pathways described above, Table 1 provides a structured synthesis of the principal mechanisms governing nanoparticle–membrane interactions. The table systematically links dominant interaction modes with key nanoparticle physicochemical attributes, membrane properties, and the experimental and computational approaches commonly used to interrogate them. By juxtaposing mechanistic drivers, membrane responses, and methodological strategies, this overview highlights both shared principles and critical distinctions across interaction pathways. As such, Table 1 serves as a conceptual framework that integrates molecular mechanisms with experimental observables and computational models, and it provides a foundation for interpreting the representative case studies discussed in Section 6.2.

Building on this mechanistic framework, the following case studies illustrate how specific nanoparticle designs and membrane environments translate these interaction pathways into experimentally observable behavior and functional outcomes.

### 6.2. Representative Case Studies

Four scenarios exemplify how nanoscale design characteristics influence membrane behavior at bio–nano interfaces. They all tie together physicochemical attributes with the evident transformations in membrane design, kinetics, and capabilities.

Gold nanoparticles (AuNP) with Langmuir (e.g., DPPC) monolayers: Size, ligand chemistry, and hydrophobicity are the primary determinants of AuNP–DPPC interactions. Particles ~5 nm in diameter can penetrate and progressively reorganize the monolayer over time [186]. Hydrophilic ligands facilitate traversal of the headgroup region, whereas hydrophobic ligands embed within the film—particularly under compression—thereby altering its structure [187]. Hydrophobic AuNPs also perturb the phase behavior and mechanical properties of the monolayer, modifying elasticity and surface tension, and shifting collapse conditions relevant to respiratory cycling [188,189]. These effects present a biomedical duality because they may enhance pulmonary drug delivery but also risk compromising surfactant function [187,190].

Polymer-coated NPs with SLBs: Polymer–lipid hybrids enable efficient loading of diverse therapeutics, including nucleic acids and proteins [191], with lipid–polymer nanoparticles improving payload capacity and release for targeted cancer applications [192]. SLBs formed on nanoparticle arrays maintain lateral mobility and structural integrity and, when combined with flat regions, permit curvature-dependent studies of membrane phenomena [87]. Amphiphilic polymer design further tunes membrane engagement and translocation, with specific copolymer patterns engineered to optimize bilayer penetration [193].

Nanostructured peptides forming pores: The cytoplasmic membrane is a primary target of antimicrobial peptides (AMPs), so clarifying their molecular mechanisms is crucial for designing new membrane-specific agents. In membrane-active peptides, nanoscale pore formation, namely its structure, size, and stability, governs membrane disruption and loss of cellular homeostasis, often representing the final step before cell death; accordingly, biophysical methods with nanoscale resolution are essential to resolve these processes [184]. Amphipathic α-helical scaffolds (e.g., melittin derivatives) insert into lipid bilayers and assemble pores stabilized by cooperative hydrogen-bond networks with lipid headgroups. Macrolittins exemplify this behavior, forming selective, stable pores with reduced cytotoxicity [194]. D-amino-acid peptides such as DpPorA exhibit proteolytic stability and form cation-selective pores via characteristic α-helical packing and surface charge [185], although pore size and lifetime remain variable, motivating further mechanistic study [195].

Toxicological aspects and NP-induced membrane leakage: Lysosomal accumulation can trigger membrane rupture/permeabilization and the “lysosome-enhanced Trojan horse effect,” where acidic compartments drive ion release [196,197]. Ion dissolution and cationic NP–membrane interactions promote oxidative stress and inflammation [197,198]. Not all NPs are equivalently hazardous; stable, non-ion-releasing or robustly coated formulations show reduced toxicity, underscoring the role of surface chemistry in safe-by-design strategies [197].

In summary, these cases reveal significant design criteria—measurements, electrostatic forces, ligand orientation, and peptide secondary arrangement—that govern the level to which membranes face remodeling, incursion, or destabilization. The evolution of these mechanistic findings into calculable design doctrines can integrate therapeutic advantages with safety in significant biological realms.

## 7. Applications, Current Challenges, and Future Perspectives

**Figure 15:**
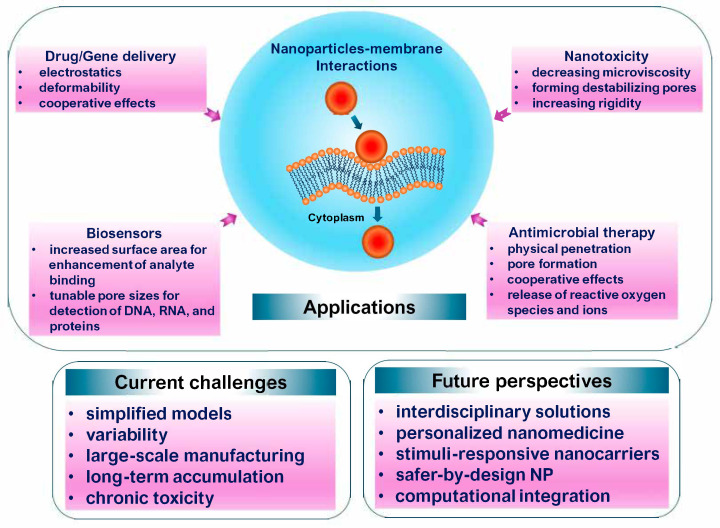
Applications, challenges, and future perspectives of nanoparticle–membrane interactions. This original schematic illustrates major biomedical applications (drug/gene delivery, antimicrobial therapy, biosensors, and nanotoxicology), current challenges (simplified models, variability, large-scale manufacturing, long-term accumulation, and chronic toxicity), and future directions (interdisciplinary approaches, personalized nanomedicine, stimuli-responsive nanocarriers, safer-by-design strategies, and computational integration) (Original schematic).

A conceptual overview of current applications, technical challenges, and future directions is encapsulated in Figure 15, providing a structural framework for the advancement of safe and effective nanomedicines. Nanoparticle–membrane interactions underpin a broad spectrum of therapeutic delivery strategies, in which controlled adsorption, partial insertion, or membrane remodeling govern cellular uptake and subsequent intracellular trafficking [33,191]. Recent mechanistic studies further demonstrate that membrane lipid composition and nanoparticle surface chemistry act cooperatively to regulate translocation efficiency and endocytic routing, with subtle variations in interfacial charge distribution or lipid packing leading to pronounced differences in delivery outcomes [167,168].

The efficacy of nanoparticle–membrane interactions derives from physicochemical attributes such as size, charge, shape, elasticity, and surface functionalization, which govern engagement with lipid bilayers and cellular barriers [138,199]. In drug and gene delivery, cationic nanoparticles bind strongly to lipid bilayers and promote penetration [138], while anionic particles interact more weakly but modulate bilayer diffusivity. Geometry also matters, i.e., oblate nanoparticles display enhanced uptake compared to rigid spheres due to increased deformability [183]. Partner-assisted uptake further illustrates cooperative effects [200]. Because hydrophobic bilayers restrict hydrophilic drug permeability [183], electroporation and cell-penetrating peptides are often exploited to enhance cytosolic delivery [201]. Importantly, many therapeutics rely on transporter-mediated uptake rather than passive diffusion, emphasizing the need to align nanocarrier design with membrane protein biology [202].

Antimicrobial nanoparticles offer complementary benefits by targeting bacterial membranes. Membrane disruption, pore formation, and lipid extraction have been identified as key mechanisms underlying both antimicrobial efficacy and cytotoxic responses [196,197]. Physical penetration and pore formation compromise structural integrity [203,204], while metallic nanoparticles such as silver and zinc oxide (ZnO) generate reactive oxygen species and release ions that further disrupt membranes and intracellular processes [205,206]. These features make them effective against biofilms [207], valuable in device coatings [208], and synergistic with antibiotics [209]. Nonetheless, cost-effective production, physicochemical heterogeneity, and cytotoxicity remain major barriers [204].

Biosensor technologies likewise exploit nanoporous and model membranes to achieve high sensitivity and specificity. The integration of lipid membranes into biosensor platforms improves molecular selectivity while reducing biofouling, enabling highly sensitive and biologically relevant detection schemes [210,211]. Increased surface area facilitates analyte binding, while nanopore architectures amplify signals [212,213]. Their adaptability enables integration with electrochemical and optical platforms [214], and tunable pore sizes allow detection of DNA, RNA, and proteins [215]. The use of natural membranes further reduces biofouling and enhances molecular recognition [210]. Remaining obstacles include multiplexed detection and the optimization of membrane materials [211].

At the same time, nanoparticle–membrane interactions pose risks relevant to nanotoxicology. Small nanoparticles (<10 nm) penetrate bilayers, decreasing microviscosity and forming destabilizing pores [216], while larger ones adsorb at the surface, increasing rigidity and altering bilayer organization. Both behaviors disrupt lipid phase transitions and vesicle stability [217]. Oxidative stress, a central mechanism of toxicity, arises from reactive oxygen species that damage lipids, proteins, and DNA, triggering inflammatory cascades [198]. Toxicological outcomes strongly depend on nanoparticle size, shape, charge, and surface chemistry, which may transform in biological environments [31,48]. Thermodynamic analyses further show that such perturbations can alter signaling pathways and gene expression [218,219].

Despite evident opportunities, critical challenges persist. The formation of membrane-mediated ordered structures remains difficult to engineer, yet such architectures could substantially enhance signal transduction and molecular recognition in biosensing applications. The formation of stable nanoscale domains with controlled composition and geometry requires precise regulation of lipid–protein interactions, curvature, and lateral phase behavior [27,97]. Although biomimetic and hybrid membrane platforms can reproduce aspects of biological ordering, the reliable engineering of raft-like architectures or asymmetric distributions remains limited by the sensitivity of domain stability to lipid composition and environmental conditions [27,98]. Nevertheless, ordered architectures have demonstrated the capacity to amplify signal transduction, enhance sensitivity, and support multiplexed detection, positioning them as promising but underdeveloped tools for membrane-based sensing technologies [27].

The construction of lipid nanoparticles (LNPs) and related supramolecular assemblies presents parallel challenges due to their multicomponent composition, dynamic structure, and complex spatial organization. Small changes in lipid ratios, hydration conditions, or fabrication protocols can alter nanoparticle size, colloidal stability, and encapsulation efficiency, complicating reproducibility and functional optimization [42,46]. These sensitivities are particularly problematic for drug delivery and nanotoxicology, where membrane rigidity, surface charge, and lipid mixing dynamics strongly influence nanoparticle uptake, immune recognition, and biodistribution [43,44]. From a computational perspective, realistic modeling of LNPs remains constrained by conformational heterogeneity and large time- and length-scale requirements, limiting the predictive capacity of simulations for structure–function relationships [6,26,62]. These barriers highlight the need for tighter experimental–computational integration to support rational nanocarrier design.

Addressing these challenges is central not only to biosensor performance, but also to the safety and efficacy of nanomedicines. The capacity to design nanocarriers that account for membrane heterogeneity, physiological variability, and immune signaling will be essential for mitigating chronic toxicity and improving clinical translation. In this regard, personalized nanomedicine—tailoring nanoparticle composition, surface chemistry, and delivery mechanisms to patient-specific membrane phenotypes—represents a compelling direction for future research, yet remains in its early stages.

Looking ahead, reconciling efficacy with safety will require interdisciplinary strategies. Computational integration, particularly the combination of multiscale molecular dynamics with machine learning, enables accelerated exploration of nanoparticle design space, improved predictive accuracy, and reduced simulation cost [6,26,220]. Environmentally, safer-by-design nanoparticles and green nanotechnologies will be crucial to mitigate ecological risks. The development of predictive frameworks that bridge experimental, computational, and in vivo approaches is therefore essential for translating nanoscale stability and interfacial behavior into clinically relevant outcomes.

## 8. Conclusions

This review synthesizes how nanoparticles and molecular nanostructures engage model membrane systems across scales—from thermodynamic signatures at the air–water interface to atomistic pathways of permeation. Through the comparative analysis of polymeric, lipid-based, peptide/dendrimer, and hybrid constructs on Langmuir monolayers, supported lipid bilayers, liposomes/vesicles, and intricate biomimetic membranes, consistent mechanistic pathways become apparent: surface adsorption, bilayer insertion, pore formation, and lipid reorganization influenced by particle size/shape, surface charge, ligand architecture, as well as by membrane composition, phase state, curvature, and asymmetry. A comprehensive multitechnique toolkit (Langmuir troughs and BAM; AFM and QCM-D; fluorescence spectroscopy/microscopy; NR/XRR; and MD simulations) yields complementary data that, when integrated, elucidate the phenomena occurring at the interface, the velocity of these processes, their penetration depth, and the specific conditions under which they transpire.

These descriptors are directly applicable to various domains. In the fields of pharmacological and genetic delivery, electrostatic forces and the lipophilicity of ligands modulate adsorption characteristics and penetration efficacy, whereas alterations in membrane architecture are associated with the accessibility of cargo and the successful escape from endosomal compartments. In the context of antimicrobial design, peptide-mediated pore formation and the utilization of membrane-active nanoparticles take advantage of curvature and packing irregularities to disrupt bacterial membranes. The sensitivity and specificity in biosensing are collectively influenced by membrane integrity, domain organization, and the accuracy of functionalization. The discipline of nanotoxicology concurrently unveils potential hazards—such as ion leaching, oxidative stress, lysosomal destabilization, and chronic accumulation—subject to modulation by the dynamic identities of particles (including protein and environmental coronas) and the properties of the interacting membranes.

The unification of straightforward in vitro and in silico models with the elaborate workings of in vivo systems still deals with pronounced difficulties. Advancements in this area will be enhanced by (i) the development of hybrid mimetics that encompass proteins, asymmetric lipid compositions, and meticulously controlled curvatures; (ii) the establishment of standardized protocols for the reporting of composition, dosing, and interfacial metrics to facilitate reproducibility and comparability; and (iii) the implementation of correlative pipelines that harmonize conditions across Langmuir/BAM, SLB AFM–QCM-D, vesicle fluorescence, and calibrated molecular dynamics, with an increasing reliance on machine learning methodologies. The incorporation of these practices within frameworks that prioritize safety-by-design and manufacturing considerations will ensure that mechanistic assays are in alignment with regulatory standards. This integration allows interfacial measurements to transition from descriptive observations into predictive models, enabling the creation of nanostructures that are both mechanistically detailed and applicable in therapy, diagnostics, and for promoting environmental sustainability.

## Figures and Tables

**Table 1:** Mechanisms of nanoparticle–membrane interactions: physicochemical drivers, membrane response, and methodological approaches.

Interaction Mechanism	NP Properties	Membrane Properties	Typical Experimental Methods	Computational Approaches	Key Outcomes	Refs.
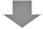						
**Adsorption**	Surface charge (cationic > anionic); hydrophobicity; surface chemistry; functional groups (amine, carboxyl)	Surface charge density; lipid head group composition; membrane fluidity; presence of cholesterol	Fluorescence microscopy; surface plasmon resonance (SPR); quartz crystal microbalance with dissipation monitoring (QCM-D); zeta potential; isothermal titration calorimetry (ITC); dynamic light scattering (DLS)	Molecular dynamics (MD) simulations; coarse-grained models; free energy calculations; umbrella sampling	Initial interfacial contact; electrostatic and hydrophobic anchoring; largely reversible binding; prerequisite for subsequent insertion or translocation	[31,33,168]
**Partial** **insertion**	Moderate hydrophobicity; size (typically <10 nm; mechanism-dependent); shape (spherical or short-rod; aspect ratio–dependent); surface ligand density and length	Lipid tail order; membrane thickness; phase state (liquid-disordered favors insertion); lipid saturation	Fluorescence quenching; electron paramagnetic resonance (EPR); nuclear magnetic resonance (NMR); neutron reflectometry; Förster resonance energy transfer (FRET); cryo-electron microscopy (cryo-EM)	Atomistic MD; potential of mean force (PMF) calculations; enhanced sampling methods	NP penetrates outer leaflet; lipid tail disordering; local membrane thinning; increased permeability; partial hydrophobic matching	[167,168,169]
**Full** **translocation**	Small size (<5 nm); amphiphilic character; moderate surface charge; ligand flexibility; core composition	Low bending rigidity; high fluidity; thin membranes; asymmetric lipid distribution; low cholesterol	Single-particle tracking; confocal microscopy; flow cytometry; transmission electron microscopy (TEM); mass spectrometry	Steered MD; free energy perturbation; transition path sampling; metadynamics	Complete membrane crossing; transient pore formation; energy barrier overcome; size-dependent kinetics; limited long-term membrane disruption (system-dependent)	[165,168,169]
**Pore** **formation**	High positive charge; rigid structure; size 2–10 nm; cylindrical/rod shape; high aspect ratio	Low line tension; presence of anionic lipids; membrane tension; lipid packing defects	Patch-clamp electrophysiology; conductance measurements; leakage assays (calcein, carboxyfluorescein); atomic force microscopy (AFM); planar lipid bilayers	Coarse-grained MD; pore nucleation simulations; continuum elasticity models; free energy landscapes	Transient or stable aqueous pores; ion leakage; loss of membrane potential; cell death pathway; cooperative NP assembly at pore edge	[8,184,185]
**Lipid** **extraction**	Hydrophobic surface; high curvature; small size; surface roughness; aromatic groups	Lipid packing density; presence of unsaturated lipids; membrane defects; lipid flip-flop rate	Lipidomics; mass spectrometry; lipid binding assays; fluorescence correlation spectroscopy (FCS); laurdan generalized polarization (GP)	Atomistic MD; lipid transfer free energy; contact angle analysis; hydrophobic interaction quantification	Individual lipid molecules extracted; membrane thinning; increased permeability; lipid composition changes; cooperative extraction events	[32,173,178]
**Endocytosis-like wrapping**	Size (20–50 nm optimal); shape (spherical preferred); surface ligand type and density; receptor-binding capability	Membrane bending modulus; receptor density; cortical tension; actin coupling; clathrin availability	Live-cell imaging; TEM; receptor blocking studies; dynamin inhibition; clathrin knockdown; correlative light-electron microscopy (CLEM)	Continuum membrane models; hybrid continuum–particle simulations; receptor-mediated adhesion models; shape evolution calculations; energy minimization	Progressive membrane curvature; receptor clustering; clathrin coat assembly; vesicle budding; energy-driven process; size/shape selectivity	[101,102,177]

Mechanisms of nanoparticle–membrane interactions: physicochemical drivers, membrane response, and methodological approaches. The table synthesizes six primary nanoparticle–membrane interaction mechanisms, correlating governing physicochemical parameters with membrane responses and methodological approaches. Abbreviations: NP, nanoparticle; SPR, surface plasmon resonance; QCM-D, quartz crystal microbalance with dissipation monitoring; ITC, isothermal titration calorimetry; DLS, dynamic light scattering; MD, molecular dynamics; EPR, electron paramagnetic resonance; NMR, nuclear magnetic resonance; FRET, Förster resonance energy transfer; cryo-EM, cryo-electron microscopy; PMF, potential of mean force; TEM, transmission electron microscopy; AFM, atomic force microscopy; FCS, fluorescence correlation spectroscopy; GP, generalized polarization; CLEM, correlative light-electron microscopy.

## Data Availability

No new data were created or analyzed in this study.

## Abbreviations

The following abbreviations are used in this manuscript:

AFM	Atomic Force Microscopy
ALEX	Alternating-Laser Excitation
AMPs	Antimicrobial Peptides
AuNPs	Gold Nanoparticles
BAM	Brewster Angle Microscopy
CG-MD	Coarse-Grained Molecular Dynamics
CLSM	Confocal Laser Scanning Microscopy
CRediT	Contributor Roles Taxonomy
DNA	Deoxyribonucleic Acid
DPPC	Dipalmitoylphosphatidylcholine
FRET	Förster Resonance Energy Transfer
FRAP	Fluorescence Recovery After Photobleaching
GIXD	Grazing-Incidence X-ray Diffraction
GUVs	Giant Unilamellar Vesicles
HBMs	Hybrid Bilayer Membranes
HMEC-1	Human Microvascular Endothelial Cells (line 1)
IRRAS	Infrared Reflection–Absorption Spectroscopy
LUVs	Large Unilamellar Vesicles
MD	Molecular Dynamics
MLVs	Multilamellar Vesicles
MRFS	Molecular Recognition Force Spectroscopy
MVVs	Multivesicular Vesicles
NPs	Nanoparticles
NR	Neutron Reflectometry
PBCA	Poly(Butyl Cyanoacrylate)
PEGylation	Poly(ethylene glycol) surface modification
PLA_2_	Phospholipase A_2_
PNPs	Polymeric Nanoparticles
QCM-D	Quartz Crystal Microbalance with Dissipation monitoring
RNA	Ribonucleic Acid
SAM	Self-Assembled Monolayer
SEIRAS	Surface-Enhanced Infrared Absorption Spectroscopy
SLBs	Supported Lipid Bilayers
SLNs	Solid Lipid Nanoparticles
smFRET	Single-Molecule Förster Resonance Energy Transfer
STED	Stimulated Emission Depletion (Microscopy)
SUVs	Small Unilamellar Vesicles
XR	X-ray Reflectivity
XRR	X-ray Reflectometry

## References

[1] Ferrari E., Soloviev M. (2012). Nanoparticles in Biology and Medicine.

[2] Afzal O., Altamimi A.S.A., Nadeem M.S., Alzarea S.I., Almalki W.H., Tariq A., Mubeen B., Murtaza B.N., Iftikhar S., Riaz N. (2022). Nanoparticles in Drug Delivery: From History to Therapeutic Applications. Nanomaterials.

[3] Rather M., Muzamil S., Nabi S., Salahi A., Zhang J., Prasad M. (2022). Application of Nanotechnology in Drug Delivery.

[4] Hamad-Schifferli K. (2023). Nano-Bio Interfaces in Colloids and Nanoparticles. One Hundred Years of Colloid Symposia: Looking Back and Looking Forward.

[5] Wang Y., Cai R., Chen C. (2019). The Nano–Bio Interactions of Nanomedicines: Understanding the Biochemical Driving Forces and Redox Reactions. Acc. Chem. Res..

[6] Marrink S.J., Corradi V., Souza P.C., Ingolfsson H.I., Tieleman D.P., Sansom M.S. (2019). Sansom Computational Modeling of Realistic Cell Membranes. Chem. Rev..

[7] Brown C.M., Marrink S.J. (2024). Modeling Membranes in Situ. Curr. Opin. Struct. Biol..

[8] Kariuki R., Mirihana K.A., Penman R., Hung A., Meftahi N., Bryant G., Ramsland P.A., Voïtchovsky K., Conn C.E., Contini C. (2025). Interactions of Nanoparticles with Living and Synthetic Bio-Membranes. Chem. Soc. Rev..

[9] Barua S., Chen B., Baruah S. (2019). Baruah Bio-Nano Interfacial Interactions for Drug Delivery Systems. Dynamics of Advanced Sustainable Nanomaterials and Their Related Nanocomposites at the Bio-Nano Interface.

[10] Singh R., Long F.R., Saini A., Joma N., Basu A., Mahmoudi M., Vali H., Kakkar A. (2025). Nano-Bio Interactions and Drug Delivery Using Soft Nanoparticles: A New Paradigm in Pharmaceutical Cargo Release. RSC Pharm..

[11] Veg E., Hashmi K., Raza S., Joshi S., Rahman Khan A., Khan T. (2025). The Role of Nanomaterials in Diagnosis and Targeted Drug Delivery. Chem. Biodivers..

[12] Abughalia A., Flynn M., Clarke P.F.A., Fayne D., Gobbo O.L. (2025). The Use of Computational Approaches to Design Nanodelivery Systems. Nanomaterials.

[13] Oreopoulos J. (2011). Combinatorial Microscopy of Molecular Interactions at Membrane Interfaces.

[14] Pelaz B., Charron G., Pfeiffer C., Zhao Y., De La Fuente J.M., Liang X.J., Parak W.J., Del Pino P. (2013). Interfacing Engineered Nanoparticles with Biological Systems: Anticipating Adverse Nano-Bio Interactions. Small.

[15] Stillwell W. (2013). An Introduction to Biological Membranes: From Bilayers to Rafts.

[16] Eeman M., Deleu M. (2010). From Biological Membranes to Biomimetic Model Membranes. Biotechnol. Agron. Société Environ..

[17] Piacentini E., Mazzei R., Drioli E., Giorno L. (2017). 1.1 From Biological Membranes to Artificial Biomimetic Membranes and Systems.

[18] Rothstein A. (1984). Membrane Mythology: Technical versus Conceptual Developments in the Progress of Research. Can. J. Biochem. Cell Biol..

[19] Mc Cabe I.P. (2015). Observing, Understanding, and Manipulating Biological Membranes. Ph.D. Thesis.

[20] Keller H., Worch R., Schwille P. (2013). Model Membrane Systems. Methods of Molecular Biology.

[21] Redwan D.A., Yong X. (2025). Recent Advances in Computational Modeling of the Effects of Micro/Nanoparticle Interactions on Vesicle Morphology. Advances in Biomembranes and Lipid Self-Assembly.

[22] Baszkin A., Norde W. (1999). Physical Chemistry of Biological Interfaces.

[23] Sezgin E., Schwille P. (2012). Model Membrane Platforms to Study Protein-Membrane Interactions. Mol. Membr. Biol..

[24] Nguyen M.H., Rickeard B.W., DiPasquale M., Marquardt D. (2019). Asymmetric Model Membranes: Frontiers and Challenges. Biomimetic Lipid Membranes: Fundamentals, Applications, and Commercialization.

[25] Shin J., Jeong S.H., Shon M.J. (2025). Advancing Membrane Biology: Single-Molecule Approaches Meet Model Membrane Systems. BMB Rep..

[26] Enkavi G., Javanainen M., Kulig W., Róg T., Vattulainen I. (2019). Multiscale Simulations of Biological Membranes: The Challenge To Understand Biological Phenomena in a Living Substance. Chem. Rev..

[27] Coronado S., Herrera J., Pino M.G., Martín S., Ballesteros-Rueda L., Cea P. (2024). Advancements in Engineering Planar Model Cell Membranes: Current Techniques, Applications, and Future Perspectives. Nanomaterials.

[28] Guo J., Bao Y., Li M., Li S., Xi L., Xin P., Wu L., Liu H., Mu Y. (2023). Application of Computational Approaches in Biomembranes: From Structure to Function. WIREs Comput. Mol. Sci..

[29] Cornet J., Coulonges N., Pezeshkian W., Penissat-Mahaut M., Desgrez-Dautet H., Marrink S.J., Destainville N., Chavent M., Manghi M. (2024). There and Back Again: Bridging Meso- and Nano-Scales to Understand Lipid Vesicle Patterning. Soft. Matter..

[30] Cortez-Jugo C., Czuba-Wojnilowicz E., Tan A., Caruso F. (2021). A Focus on “Bio” in Bio-Nanoscience: The Impact of Biological Factors on Nanomaterial Interactions. Adv. Healthc. Mater..

[31] Issler T., Turner R.J., Prenner E.J. (2024). Membrane-Nanoparticle Interactions: The Impact of Membrane Lipids. Small.

[32] Cecchet F. (2024). Light on the Interactions between Nanoparticles and Lipid Membranes by Interface-Sensitive Vibrational Spectroscopy. Colloids Surf. B Biointerfaces.

[33] Balog S., De Almeida M.S., Taladriz-Blanco P., Rothen-Rutishauser B., Petri-Fink A. (2024). Does the Surface Charge of the Nanoparticles Drive Nanoparticle–Cell Membrane Interactions?. Curr. Opin. Biotechnol..

[34] Beach M.A., Nayanathara U., Gao Y., Zhang C., Xiong Y., Wang Y., Such G.K. (2024). Polymeric Nanoparticles for Drug Delivery. Chem. Rev..

[35] Parashar K., Khan M.H. (2023). Polymeric Nanoparticles in Drug Delivery. Advances in Medical Diagnosis, Treatment, and Care (AMDTC) Book Series.

[36] Niculescu A.G., Grumezescu A.M. (2021). Polymer-Based Nanosystems—A Versatile Delivery Approach. Materials.

[37] Gillella S., Divyanjali M., Rishitha S., Amzad S.K., Reddy U., Girish C., Apparao C.H. (2024). Polymeric Nanoparticles—A Review. J. Innov. Appl. Pharm. Sci..

[38] Kenry Yeo T., Manghnani P.N., Middha E., Pan Y., Chen H., Lim C.T., Liu B. (2020). Mechanistic Understanding of the Biological Responses to Polymeric Nanoparticles. ACS Nano.

[39] Deshmukh S., Chaudhari B., Velhal A., Redasani V. (2022). A Compendious Review on Biodegradable Polymeric Nanoparticles. Asian J. Pharm. Technol..

[40] Naha A., Priya J., Dhoot A., Xalxo N. (2018). A Review on Polymeric Nanoparticles: A Promising Novel Drug Delivery System. J. Glob. Pharma Technol..

[41] Carina I.C. (2015). Crucho Stimuli-Responsive Polymeric Nanoparticles for Nanomedicine. ChemMedChem.

[42] Samimi S., Maghsoudnia N., Eftekhari R.B., Dorkoosh F., Mohapatra S.S., Ranjan S., Dasgupta N., Mishra R.K., Thomas S. (2019). Chapter 3—Lipid-Based Nanoparticles for Drug Delivery Systems. Characterization and Biology of Nanomaterials for Drug Delivery.

[43] Awale R., Kulkarni N., Khadse S. (2024). Interactions of Nanoparticles with Lipid and Cell Membranes. Mater. Res. Found..

[44] Jeitler R., Glader C., Tetyczka C., Zeiringer S., Absenger-Novak M., Selmani A., Fröhlich E., Roblegg E. (2022). Investigation of Cellular Interactions of Lipid-Structured Nanoparticles With Oral Mucosal Epithelial Cells. Front. Mol. Biosci..

[45] Jana M., Biswas U.K., Patro C.S., Debnath B., Sharma S., Naskar S. (2024). Solid Lipid Nanoparticles: A Review of Their Biomedical Applications and Preparation. Pharm. Nanotechnol..

[46] Nguyen T.T.L., Duong V.A. (2022). Solid Lipid Nanoparticles. Encyclopedia.

[47] Patel D., Patel B., Thakkar H. (2021). Lipid Based Nanocarriers: Promising Drug Delivery System for Topical Application. Eur. J. Lipid Sci. Technol..

[48] Hitler L., Nyong’a M.T., Ali I., Sadia A., Waliaula K.P., Ogalo O.J., Udochukwu A.O. (2017). Mechanism, of Biophysicochemical Interactions and Cellular Uptake at the Nano-Bio Interface: A Review. Eur. J. Biophys..

[49] Mitrovic J., Richey G., Kim S., Guler M.O. (2023). Peptide Hydrogels and Nanostructures Controlling Biological Machinery. Langmuir.

[50] Huang H., Kiick K. (2022). Peptide-Based Assembled Nanostructures That Can Direct Cellular Responses. Biomed. Mater..

[51] Song S.J., Choi J.S. (2022). Enzyme-Responsive Amphiphilic Peptide Nanoparticles for Biocompatible and Efficient Drug Delivery. Pharmaceutics.

[52] König N., Szostak S.M., Nielsen J.E., Dunbar M., Yang S., Chen W., Benjamin A., Radulescu A., Mahmoudi N., Willner L. (2023). Stability of Nanopeptides: Structure and Molecular Exchange of Self-Assembled Peptide Fibers. ACS Nano.

[53] Lai Z., Jian Q., Li G., Shao C., Zhu Y., Yuan X., Chen H., Shan A. (2021). Self-Assembling Peptide Dendron Nanoparticles with High Stability and a Multimodal Antimicrobial Mechanism of Action. ACS Nano.

[54] Maiti S., Jana S. (2017). Dendrimers as Nanostructured Therapeutic Carriers. Particulate Technology for Delivery of Therapeutics.

[55] Gu Z., Luo K., She W., Wu Y., He B. (2010). New-Generation Biomedical Materials: Peptide Dendrimers and Their Application in Biomedicine. Sci. China Chem..

[56] Zelzer M., Ulijn R.V. (2010). Next-Generation Peptide Nanomaterials: Molecular Networks, Interfaces and Supramolecular Functionality. Chem. Soc. Rev..

[57] Harris A.W., Cha J.N. (2020). Bridging Bio-Nano Interactions with Photoactive Biohybrid Energy Systems. Mol. Syst. Des. Eng..

[58] Bukhari R. (2024). Introduction to Hybrid Nanomaterial. Advances in Chemical and Materials Engineering Book Series.

[59] Zhang D., Chen Y., Hao M., Xia Y. (2024). Putting Hybrid Nanomaterials to Work for Biomedical Applications. Angew. Chem. Int. Ed..

[60] Kumar R., Kumar P. (2024). Exploring the Diverse Biomedical Applications of Hybrid Nanomaterials. Advances in Chemical and Materials Engineering Book Series.

[61] Hashim A., Fatima F. (2024). Exploring the Transformative Potential of Hybrid Nanoparticles in Biomedical Applications. Advances in Chemical and Materials Engineering Book Series.

[62] Casalini T., Limongelli V., Schmutz M., Som C., Jordan O., Wick P., Borchard G., Perale G. (2019). Molecular Modeling for Nanomaterial-Biology Interactions: Opportunities, Challenges, and Perspectives. Front. Bioeng. Biotechnol..

[63] Forte G., La Mendola D., Satriano C. (2023). The Hybrid Nano-Biointerface between Proteins/Peptides and Two-Dimensional Nanomaterials. Molecules.

[64] Umer A., Ghouri M.D., Muyizere T., Aqib R.M., Muhaymin A., Cai R., Chen C. (2023). Engineered Nano–Bio Interfaces for Stem Cell Therapy. Precis. Chem..

[65] Gómez-Gómez A.L., del Rosario Moguel-Concha D., Borges-Martínez J.E., Martínez-Ayala A.L., Dávila-Ortiz G. (2024). Bio–Nano Interface Technology for Biomedical Applications. Nanoscience and Nanotechnology for Smart Prevention, Diagnostics and Therapeutics: Fundamentals to Applications.

[66] Adamson A.W., Gast A.P. (1997). Physical Chemistry of Surfaces.

[67] Brezesinski G., Möhwald H. (2003). Langmuir Monolayers to Study Interactions at Model Membrane Surfaces. Adv. Colloid Interface Sci..

[68] Pereira A.R., Oliveira Junior O.N. (2021). Recent Advances in the Use of Langmuir Monolayers as Cell Membrane Models. Eclética Química.

[69] Dennin M. (2014). Langmuir Monolayers.

[70] Dynarowicz-Łątka P., Dhanabalan A., Oliveira O.N. (2001). Modern Physicochemical Research on Langmuir Monolayers. Adv. Colloid Interface Sci..

[71] O’Connor E. (2006). Discontinuous Molecular Dynamics Studies of Model Langmuir Monolayers. Master’s Thesis.

[72] Rojewska M., Smułek W., Kaczorek E., Prochaska K. (2021). Langmuir Monolayer Techniques for the Investigation of Model Bacterial Membranes and Antibiotic Biodegradation Mechanisms. Membranes.

[73] Dynarowicz-Latka P., Wnętrzak A., Chachaj-Brekiesz A. (2024). Advantages of the Classical Thermodynamic Analysis of Single—And Multi-Component Langmuir Monolayers from Molecules of Biomedical Importance—Theory and Applications. J. R. Soc. Interface.

[74] Siegel A.P., Naumann C.A. (2009). Polymer Stabilized Lipid Membranes: Langmuir Monolayers.

[75] Yordanov G., Minkov I., Balashev K. (2024). The Langmuir Monolayer as a Model Membrane System for Studying the Interactions of Poly(Butyl Cyanoacrylate) Nanoparticles with Phospholipids at the Air/Water Interface. Membranes.

[76] Moehwald H., Brezesinski G. (2016). From Langmuir Monolayers to Multilayer Films. Langmuir.

[77] Hąc-Wydro K., Dynarowicz-Łątka P. (2008). Biomedical Applications of the Langmuir Monolayer Technique. Ann. Umcs Chem..

[78] Sebaaly C., Greige-Gerges H., Charcosset C. (2019). Lipid Membrane Models for Biomembrane Properties’ Investigation. Current Trends and Future Developments on (Bio-)Membranes.

[79] Sondhi P., Lingden D., Stine K.J. (2020). Structure, Formation, and Biological Interactions of Supported Lipid Bilayers (SLB) Incorporating Lipopolysaccharide. Coatings.

[80] Cava D.G., Vélez M. (2022). Vélez Supported Lipid Bilayers (SLBs) to Study Amyloid-Lipid Membrane Interactions with Atomic Force Microscopy. Bacterial Amyloids: Methods and Protocols.

[81] Morandat S., Kirat K.E. (2010). Exploring the Properties and Interactions of Supported Lipid Bilayers on the Nanoscale by Atomic Force Microscopy. Microsc. Sci. Technol. Appl. Educ..

[82] Sut T.N., Park S., Choe Y., Cho N.J. (2019). Characterizing the Supported Lipid Membrane Formation from Cholesterol-Rich Bicelles. Langmuir.

[83] Ahmed S., Savarala S., Chen Y., Bothun G., Wunder S.L. (2012). Formation of Lipid Sheaths around Nanoparticle-Supported Lipid Bilayers. Small.

[84] Xia Z., Woods A., Quirk A., Burgess I.J., Lau B.L.T. (2019). Interactions between Polystyrene Nanoparticles and Supported Lipid Bilayers: Impact of Charge and Hydrophobicity Modification by Specific Anions. Environ. Sci. Nano.

[85] Melby E.S., Mensch A.C., Lohse S.E., Hu D., Orr G., Murphy C.J., Hamers R.J., Pedersen J.A. (2016). Formation of Supported Lipid Bilayers Containing Phase-Segregated Domains and Their Interaction with Gold Nanoparticles. Environ. Sci. Nano.

[86] Balashev K. (2014). Atomic Force Microscopy (AFM) with Applications Spanning from Nanoscale (Bio)Catalysis and Characterization of Molecularly Ordered Nanostructures to Molecular Biology. Ph.D. Thesis.

[87] Paracini N., Gutfreund P., Welbourn R., Francisco Gonzalez-Martinez J., Zhu K., Miao Y., Yepuri N., Darwish T.A., Garvey C., Waldie S. (2023). Structural Characterization of Nanoparticle-Supported Lipid Bilayer Arrays by Grazing Incidence X-Ray and Neutron Scattering. ACS Appl. Mater. Interfaces.

[88] Akbarzadeh A., Rezaei-Sadabady R., Davaran S., Joo S.W., Zarghami N., Hanifehpour Y., Samiei M., Kouhi M., Nejati-Koshki K. (2013). Liposome: Classification, Preparation, and Applications. Nanoscale Res. Lett..

[89] Huang L., Teng W., Cao J., Wang J. (2022). Liposomes as Delivery System for Applications in Meat Products. Foods.

[90] Mora N.F. (2016). Lopez Giant Unilamellar Vesicles : An Efficient Membrane Biophysical Tool and Its Application in Drug Delivery Studies. Ph.D. Thesis.

[91] Fernandez A., Krishna J., Anson F., Dinsmore A.D., Thayumanavan S. (2022). Consequences of Noncovalent Interfacial Contacts between Nanoparticles and Giant Vesicles. Angew. Chem..

[92] Wesołowska O., Michalak K., Maniewska J., Hendrich A. (2009). Giant Unilamellar Vesicles—A Perfect Tool to Visualize Phase Separation and Lipid Rafts in Model Systems. Acta Biochim. Pol..

[93] Waeterschoot J., Gosselé W., Alizadeh Zeinabad H., Lammertyn J., Koos E., Casadevall i Solvas X. (2023). Formation of Giant Unilamellar Vesicles Assisted by Fluorinated Nanoparticles (Adv. Sci. 34/2023). Adv. Sci..

[94] HUzun H.D., Tiris Z., Czarnetzki M., López-Marqués R.L., Günther Pomorski T. (2024). Electroformation of Giant Unilamellar Vesicles from Large Liposomes. Eur. Phys. J.-Spec. Top..

[95] Raneva V., Ivanova T.Z., Verger R., Panaiotov I. (1995). Comparative Kinetics of Phospholipase A2 Action on Liposomes and Monolayers of Phosphatidylcholine Spread at the Air-Water Interface. Colloids Surf. B Biointerfaces.

[96] Jain P., Rauer S.B., Möller M., Singh S. (2022). Mimicking the Natural Basement Membrane for Advanced Tissue Engineering. Biomacromolecules.

[97] Zeng T., Gautam R.P., Ko D.H., Wu H.-L., Hosseini A., Li Y., Barile C.J., Tse E.C.M. (2022). Hybrid Bilayer Membranes as Platforms for Biomimicry and Catalysis. Nat. Rev. Chem..

[98] Puiggalí-Jou A., Del Valle L.J., Alemán C. (2019). Biomimetic Hybrid Membranes: Incorporation of Transport Proteins/Peptides into Polymer Supports. Soft. Matter..

[99] Gordano A. (2024). Hybrid Inorganic Membranes. Current Trends and Future Developments on (Bio-)Membranes.

[100] Go Y.K., Leal C. (2021). Polymer Lipid Hybrid Materials. Chem. Rev..

[101] Chugh V., Vijaya Krishna K., Pandit A. (2021). Cell Membrane-Coated Mimics: A Methodological Approach for Fabrication, Characterization for Therapeutic Applications, and Challenges for Clinical Translation. ACS Nano.

[102] Malinick A.S., Stuart D.D., Lambert A.S., Cheng Q. (2023). Curved Membrane Mimics for Quantitative Probing of Protein-Membrane Interactions by Surface Plasmon Resonance. ACS Appl. Mater. Interfaces.

[103] Mohwald H. (1990). Phospholipid and Phospholipid-Protein Monolayers at the Air/Water Interface. Annu. Rev. Phys. Chem..

[104] Giner-Casares J.J., Brezesinski G., Möhwald H. (2014). Langmuir Monolayers as Unique Physical Models. Curr. Opin. Colloid Interface Sci..

[105] Lundgren A., Agnarsson B., Zirbs R., Zhdanov V.P., Reimhult E., Hook F. (2016). Nonspecific Colloidal-Type Interaction Explains Size-Dependent Specific Binding of Membrane-Targeted Nanoparticles. ACS Nano.

[106] Chaudhury A., Debnath K., Bu W., Jana N.R., Basu J.K. (2021). Penetration and Preferential Binding of Charged Nanoparticles to Mixed Lipid Monolayers: Interplay of Lipid Packing and Charge Density. Soft. Matter..

[107] Ábrahám N., Csapó E., Bohus G., Dékány I. (2014). Interaction of Biofunctionalized Gold Nanoparticles with Model Phospholipid Membranes. Colloid Polym. Sci..

[108] Caselli L., Ridolfi A., Mangiapia G., Maltoni P., Moulin J.F., Berti D., Steinke N.J., Gustafsson E., Nylander T., Montis C. (2021). Interaction of Nanoparticles with Lipid Films: The Role of Symmetry. ChemRxiv.

[109] Peetla C., Stine A., Labhasetwar V. (2009). Biophysical Interactions with Model Lipid Membranes: Applications in Drug Discovery and Drug Delivery. Mol. Pharm..

[110] Ridolfi A., Caselli L., Montis C., Mangiapia G., Berti D., Brucale M., Valle F. (2020). Gold Nanoparticles Interacting with Synthetic Lipid Rafts: An AFM Investigation. J. Microsc..

[111] Kato T., Tatehana A., Suzuki N., Iimura K., Araki T., Iriyama K. (1995). Development of a Precisely Temperature-Programmable Langmuir Trough for Measuring Properties of Insoluble Monolayers at the Water Surface as Functions of Temperature. Jpn. J. Appl. Phys..

[112] Azam U. (2018). Investigation of Interaction of Soft Nanoparticles Based Vesicles with Lung Surfactant via Langmuir-Blodgett Trough and Quartz Crystal Microbalance Study. J. Nanomed. Nanotechnol..

[113] Moya Betancourt S.N., Cámara C.I., Riva J.S. (2023). Interaction between Pharmaceutical Drugs and Polymer-Coated Fe3O4 Magnetic Nanoparticles with Langmuir Monolayers as Cellular Membrane Models. Pharmaceutics.

[114] Villanueva M.E., Salas S.D., Vico R.V. (2023). Vico Role of the Nanoparticle Core and Capping on the Interaction with Lipid Monolayers. Advances in Biomembranes and Lipid Self-Assembly.

[115] Peetla C., Labhasetwar V. (2009). Effect of Molecular Structure of Cationic Surfactants on Biophysical Interactions of Surfactant-Modified Nanoparticles with a Model Membrane and Cellular Uptake. Langmuir.

[116] Kim J.Y., Raja S., Stellacci F. (2011). Evolution of Langmuir Film of Nanoparticles Through Successive Compression Cycles. Small.

[117] Keith J. (2012). Stine Brewster Angle Microscopy.

[118] Vollhardt D. (2014). Brewster Angle Microscopy: A Preferential Method for Mesoscopic Characterization of Monolayers at the Air/Water Interface. Curr. Opin. Colloid Interface Sci..

[119] Tabe Y., Yokoyama H. (1995). Fresnel Formula for Optically Anisotropic Langmuir Monolayers: An Application to Brewster Angle Microscopy. Langmuir.

[120] Hoenig D., Moebius D. (1991). Direct Visualization of Monolayers at the Air-Water Interface by Brewster Angle Microscopy. J. Phys. Chem..

[121] Hoffmann F., Stine K.J., Hühnerfuss H. (2005). Appearance and Disappearance of Dendritic and Chiral Patterns in Domains of Langmuir Monolayers Observed with Brewster Angle Microscopy. J. Phys. Chem. B.

[122] Rodríguez Patino J.M., Sánchez C.C., Rodríguez Niño M.R. (1999). Morphological and Structural Characteristics of Monoglyceride Monolayers at the Air−Water Interface Observed by Brewster Angle Microscopy. Langmuir.

[123] Miñones J., Dynarowicz-Łatka P., Seoane R., Iribarnegaray E., Casas M. (2004). Brewster Angle Microscopy Studies of the Morphology in Dipalmitoyl Phosphatidyl Glycerol Monolayers Spread on Subphases of Different pH. Prog. Colloid Polym. Sci..

[124] Tsao M.-W., Fischer T.M., Knobler C.M. (1995). Quantitative Analysis of Brewster-Angle Microscope Images of Tilt Order in Langmuir Monolayer Domains. Langmuir.

[125] Lautz C., Fischer T.M. (1999). Quantitative Brewster Angle Microscopy Measurements of Chiral Symmetry Breaking and Chiral Boundaries in Fatty Acid Langmuir Monolayers. Eur. Phys. J. B.

[126] Melzer V., Weidemann G., Vollhardt D., Brezesinski G., Wagner R., Struth B., Möhwald H. (1997). Brewster Angle Microscopy and X-Ray GID Studies of Morphology and Crystal Structure in Monolayers of N-Tetradecyl-Gamma,Delta-Dihydroxypentanoic Acid Amide. J. Phys. Chem. B.

[127] Lai P., Nathoo S., Ku T., Gill S., Azarmi S., Roa W., Loebenberg R., Prenner E.J. (2010). Real-Time Imaging of Interactions between Dipalmitoylphosphatidylcholine Monolayers and Gelatin Based Nanoparticles Using Brewster Angle Microscopy. J. Biomed. Nanotechnol..

[128] Mandal P., Noutsi P., Chaieb S. (2016). Cholesterol Depletion from a Ceramide/Cholesterol Mixed Monolayer: A Brewster Angle Microscope Study. Sci. Rep..

[129] Ala’a F., Brunet S.M., Paige M.F. (2012). A Comparison of Atomic Force Microscopy, Confocal Fluorescence Microscopy and Brewster Angle Microscopy for Characterizing Mixed Monolayer Surfactant Films. Current Microscopy Contributions to Advances in Science and Technology.

[130] Winsel K., Hönig D., Lunkenheimer K., Geggel K., Witt C. (2003). Quantitative Brewster Angle Microscopy of the Surface Film of Human Broncho-Alveolar Lavage Fluid. Eur. Biophys. J..

[131] Zhong J. (2011). From Simple to Complex: Investigating the Effects of Lipid Composition and Phase on the Membrane Interactions of Biomolecules Using in Situ Atomic Force Microscopy. Integr. Biol..

[132] Lv Z., Banerjee S., Zagorski K., Lyubchenko Y.L. (2018). Supported Lipid Bilayers for Atomic Force Microscopy Studies. Methods of Molecular Biology.

[133] Ebner A. (2015). Biomolecular Interactions Sensed with Atomic Force Microscopy. J. Phys. Chem. Biophys..

[134] Swana K.W., Nagarajan R., Camesano T.A. (2021). Atomic Force Microscopy to Characterize Antimicrobial Peptide-Induced Defects in Model Supported Lipid Bilayers. Microorganisms.

[135] Mescola A., Ragazzini G., Facci P., Alessandrini A. (2022). The Potential of AFM in Studying the Role of the Nanoscale Amphipathic Nature of (Lipo)-Peptides Interacting with Lipid Bilayers. Nanotechnology.

[136] Mingeot-Leclercq M.-P., Deleu M., Brasseur R., Dufrêne Y.F. (2008). Atomic Force Microscopy of Supported Lipid Bilayers. Nat. Protoc..

[137] Vandoolaeghe P., Rennie A.R., Campbell R.A., Thomas R.K., Höök F., Fragneto G., Tiberg F., Nylander T. (2008). Adsorption of Cubic Liquid Crystalline Nanoparticles on Model Membranes. Soft. Matter..

[138] Biswas N., Bhattacharya R., Saha A., Jana N.R., Basu J.K. (2015). Interplay of Electrostatics and Lipid Packing Determines the Binding of Charged Polymer Coated Nanoparticles to Model Membranes. Phys. Chem. Chem. Phys..

[139] Neupane S., Cordoyiannis G., Renner F.U., Losada-Pérez P. (2019). Real-Time Monitoring of Interactions between Solid-Supported Lipid Vesicle Layers and Short- and Medium-Chain Length Alcohols: Ethanol and 1-Pentanol. Biomimetics.

[140] Nielsen S.B., Otzen D.E. (2013). Quartz Crystal Microbalances as Tools for Probing Protein-Membrane Interactions. Methods of Molecular Biology.

[141] Guo H., Xing Q., Huang R., Lee D.W., Su R., Qi W., He Z. (2019). Real-Time QCM-D Monitoring of Deposition of Gold Nanorods on a Supported Lipid Bilayer as a Model Cell Membrane. ACS Omega.

[142] Frost R., Svedhem S. (2013). Characterization of Nanoparticle-Lipid Membrane Interactions Using QCM-D. Methods of Molecular Biology.

[143] Yousefi N., Tufenkji N. (2016). Probing the Interaction between Nanoparticles and Lipid Membranes by Quartz Crystal Microbalance with Dissipation Monitoring. Front. Chem..

[144] Lu Y., Zhang H., Wang Z., Jeong S., Jo M.-C., Park M.-H., Duan X. (2019). Real-Time Detection of Nanoparticles Interaction with Lipid Membranes Using an Integrated Acoustical and Electrical Multimode Biosensor. Part. Part. Syst. Charact..

[145] Wikström A., Svedhem S., Sivignon M., Kasemo B.H. (2008). Real-Time QCM-D Monitoring of Electrostatically Driven Lipid Transfer between Two Lipid Bilayer Membranes. J. Phys. Chem. B.

[146] Flynn K.R., Martin L.L., Ackland M.L., Torriero A.A. (2016). Real-Time Quartz Crystal Microbalance Monitoring of Free Docosahexaenoic Acid Interactions with Supported Lipid Bilayers. Langmuir.

[147] Lagali N. (2013). Confocal Laser Microscopy: Principles and Applications in Medicine, Biology, and the Food Sciences.

[148] Efeoglu E., Keating M., McIntyre J., Casey A., Byrne H.J. (2015). Determination of Nanoparticle Localisation within Subcellular Organelles in Vitro Using Raman Spectroscopy. Anal. Methods.

[149] Lerner E., Barth A., Hendrix J., Ambrose B., Birkedal V., Blanchard S.C., Börner R., Sung Chung H., Cordes T., Craggs T.D. (2021). FRET-Based Dynamic Structural Biology: Challenges, Perspectives and an Appeal for Open-Science Practices. eLife.

[150] Roobala C., Ilanila I.P., Basu J.K. (2018). Applications of STED Fluorescence Nanoscopy in Unravelling Nanoscale Structure and Dynamics of Biological Systems. J. Biosci..

[151] Solovyeva D.O., Altunina A.V., Tretyak M.V., Mochalov K.E., Oleinikov V.A. (2024). Modern Methods of Fluorescence Nanoscopy in Biology. Bioorganicheskaya Khimiya.

[152] Panconi L., Euchner J., Tashev S.A., Makarova M., Herten D.P., Owen D.M., Nieves D.J. (2024). Mapping Membrane Biophysical Nano-Environments. Nat. Commun..

[153] Dorney J., Bonnier F., Garcia A., Casey A., Chambers G., Byrne H.J. (2012). Identifying and Localizing Intracellular Nanoparticles Using Raman Spectroscopy. Analyst.

[154] Als-Nielsen J., McMorrow D. (2011). Elements of Modern X-Ray Physics.

[155] Peters G.H., Toxvaerd S., Larsen N.B., Bjørnholm T., Schaumburg K., Kjaer K. (1995). Structure and Dynamics of Lipid Monolayers: Implications for Enzyme Catalysed Lipolysis. Nat. Struct. Biol..

[156] Stefaniu C., Brezesinski G., Möhwald H. (2014). Langmuir Monolayers as Models to Study Processes at Membrane Surfaces. Adv. Colloid Interface Sci..

[157] Kjaer K., Als-Nielsen J., Helm C.A., Laxhuber L.A., Möhwald H. (1987). Ordering in Lipid Monolayers Studied by Synchrotron X-Ray Diffraction and Fluorescence Microscopy. Phys. Rev. Lett..

[158] Kjaer K. (1994). Some Simple Ideas on X-Ray Reflection and Grazing-Incidence Diffraction from Thin Surfactant Films. Phys. B Condens. Matter.

[159] Armanious A., Gerelli Y., Micciulla S., Pace H.P., Welbourn R.J., Sjoberg M., Agnarsson B., Hook F. (2022). Probing the Separation Distance between Biological Nanoparticles and Cell Membrane Mimics Using Neutron Reflectometry with Sub-Nanometer Accuracy. J. Am. Chem. Soc..

[160] Eells R., Hoogerheide D.P., Kienzle P.A., Loesche M., Majkrzak C.F., Heinrich F. (2019). Structural Investigations of Membrane-Associated Proteins by Neutron Reflectometry. Characterization of Biological Membranes: Structure and Dynamics.

[161] Gayen S., Sanyal M.K., Wolff M. (2017). Neutron Reflectivity to Characterize Nanostructured Films. Magnetic Characterization Techniques for Nanomaterials.

[162] Maximilian W.A. (2019). Skoda Recent Developments in the Application of X-Ray and Neutron Reflectivity to Soft Matter Systems. Curr. Opin. Colloid Interface Sci..

[163] Jameson C.J., Oroskar P., Song B., Yuan H., Murad S. (2019). Molecular Dynamics Studies of Nanoparticle Transport Through Model Lipid Membranes. Biomimetic Lipid Membranes: Fundamentals, Applications, and Commercialization.

[164] Chu H., Zhang Y., Li Y., Li G. (2018). Computer Simulations to Explore Membrane Organization and Transport. Membrane Biophysics: New Insights and Methods.

[165] Zhang P., Jiao F., Wu L., Kong Z., Hu W., Liang L., Zhang Y. (2022). Molecular Dynamics Simulation of Transport Mechanism of Graphene Quantum Dots through Different Cell Membranes. Membranes.

[166] Van Lehn R.C., Alexander-Katz A. (2015). Pathway for Insertion of Amphiphilic Nanoparticles into Defect-Free Lipid Bilayers from Atomistic Molecular Dynamics Simulations. Soft. Matter..

[167] Kesner L.A., Piskulich Z.A., Cui Q., Rosenzweig Z. (2023). Untangling the Interactions between Anionic Polystyrene Nanoparticles and Lipid Membranes Using Laurdan Fluorescence Spectroscopy and Molecular Simulations. J. Am. Chem. Soc..

[168] Huang-Zhu C.A., Sheavly J.K., Chew A.K., Patel S.J., Van Lehn R.C. (2024). Ligand Lipophilicity Determines Molecular Mechanisms of Nanoparticle Adsorption to Lipid Bilayers. ACS Nano.

[169] Marzouq A., Morgenstein L., Huang-Zhu C.A., Yudovich S., Atkins A., Grupi A., Van Lehn R.C., Weiss S. (2024). Long-Chain Lipids Facilitate Insertion of Large Nanoparticles into Membranes of Small Unilamellar Vesicles. Langmuir.

[170] Doktorova M., Khelashvili G., Brown M.F. (2023). Sampling Bottleneck in Validating Membrane Dynamics. bioRxiv.

[171] Bradley R., Radhakrishnan R. (2013). Coarse-Grained Models for Protein-Cell Membrane Interactions. Polymers.

[172] Song B. (2012). Permeation of Nanoparticles through a Model Membrane: CG-MD Simulations Studies. Ph.D. Thesis.

[173] Gkeka P., Angelikopoulos P., Sarkisov L., Cournia Z. (2014). Membrane Partitioning of Anionic, Ligand-Coated Nanoparticles Is Accompanied by Ligand Snorkeling, Local Disordering, and Cholesterol Depletion. PLOS Comput. Biol..

[174] Ganji N. (2019). Nanoparticle-Cell Membrane Interactions: Adsorption Kinetics and the Monolayer Response. Ph.D. Thesis.

[175] Alkhammash H.I., Li N., Berthier R., De Planque M.R.R. (2015). Native Silica Nanoparticles Are Powerful Membrane Disruptors. Phys. Chem. Chem. Phys..

[176] Daniel M., Řezníčková J., Mendová K. (2023). Interactions between Biomembrane Embedded Nanoparticles Mediated by Lipid Bilayer. Advances in Biomembranes and Lipid Self-Assembly.

[177] Sheavly J.K., Pedersen J.A., Van Lehn R.C. (2019). Curvature-Driven Adsorption of Cationic Nanoparticles to Phase Boundaries in Multicomponent Lipid Bilayers. Nanoscale.

[178] Canepa E., Salassi S., De Marco A.L., Lambruschini C., Odino D., Bochicchio D., Canepa F., Canale C., Dante S., Brescia R. (2020). Amphiphilic Gold Nanoparticles Perturb Phase Separation in Multidomain Lipid Membranes. Nanoscale.

[179] Zhang Y., Dahal U., Feng Z.V., Rosenzweig Z., Cui Q., Hamers R.J. (2021). Influence of Surface Ligand Molecular Structure on Phospholipid Membrane Disruption by Cationic Nanoparticles. Langmuir.

[180] De Macedo E.F., Santos N.S., Nascimento L.S., Mathey R., Brenet S., De Moura M.S., Hou Y., Tada D.B. (2022). Interaction between Nanoparticles, Membranes and Proteins: A Surface Plasmon Resonance Study. Int. J. Mol. Sci..

[181] Bigdelou P., Vahedi A., Kiosidou E., Farnoud A.M. (2020). Loss of Membrane Asymmetry Alters the Interactions of Erythrocytes with Engineered Silica Nanoparticles. Biointerphases.

[182] Nazemidashtarjandi S., Vahedi A., Farnoud A.M. (2020). Lipid Chemical Structure Modulates the Disruptive Effects of Nanomaterials on Membrane Models. Langmuir.

[183] Chen X., Tieleman D.P., Liang Q. (2018). Modulating Interactions between Ligand-Coated Nanoparticles and Phase-Separated Lipid Bilayers by Varying the Ligand Density and the Surface Charge. Nanoscale.

[184] Bertrand B., Garduño-Juárez R., Munoz-Garay C. (2021). Estimation of Pore Dimensions in Lipid Membranes Induced by Peptides and Other Biomolecules: A Review. Biochim. Biophys. Acta (BBA) Biomembr..

[185] Krishnan R.S., Jana K., Shaji A.H., Nair K.S., Das A.D., Vikraman D., Bajaj H., Kleinekathöfer U., Mahendran K.R. (2022). Assembly of Transmembrane Pores from Mirror-Image Peptides. Nat. Commun..

[186] Rogachev A.V., Novikova N.N., Kovalchuk M.V., Malakhova Y.N., Konovalov O.V., Stepina N.D., Shlyapnikova E.A., Kanev I.L., Shlyapnikov Y.M., Yakunin S.N. (2022). Permeation of Nanoparticles into Pulmonary Surfactant Monolayer: In Situ X-Ray Standing Wave Studies. Langmuir.

[187] Jiao F., Hossain S.I., Sang J., Saha S.C., Gu Y., Hughes Z.E., Gandhi N.S. (2022). Molecular Basis of Transport of Surface Functionalised Gold Nanoparticles to Pulmonary Surfactant. RSC Adv..

[188] Tatur S., Badia A. (2012). Influence of Hydrophobic Alkylated Gold Nanoparticles on the Phase Behavior of Monolayers of DPPC and Clinical Lung Surfactant. Langmuir.

[189] Guzmán E., Liggieri L., Santini E., Ferrari M., Ravera F. (2011). Effect of Hydrophilic and Hydrophobic Nanoparticles on the Surface Pressure Response of DPPC Monolayers. J. Phys. Chem. C.

[190] Kariuki R., Penman R., Bryant S.J., Orrell-Trigg R., Meftahi N., Crawford R.J., McConville C.F., Bryant G., Voïtchovsky K., Conn C.E. (2022). Behavior of Citrate-Capped Ultrasmall Gold Nanoparticles on a Supported Lipid Bilayer Interface at Atomic Resolution. ACS Nano.

[191] Luo Y., Cook A.B., Abdelmohsen L.K.E.A., Van Hest J.C.M. (2024). Polymer Vesicles and Lipid Nanoparticles. Annu. Rev. Mater. Res..

[192] Krishnamurthy S., Vaiyapuri R., Zhang L., Chan J.M. (2015). Lipid-Coated Polymeric Nanoparticles for Cancer Drug Delivery. Biomater. Sci..

[193] Zhang L., Becton M., Wang X. (2015). Designing Nanoparticle Translocation through Cell Membranes by Varying Amphiphilic Polymer Coatings. J. Phys. Chem. B.

[194] Rangubpit W., Sungted S., Wong-Ekkabut J., Distaffen H.E., Nilsson B.L., Dias C.L. (2024). Pore Formation by Amyloid-like Peptides: Effects of the Nonpolar–Polar Sequence Pattern. ACS Chem. Neurosci..

[195] Lipkin R., Lazaridis T. (2017). Computational Studies of Peptide-Induced Membrane Pore Formation. Philos. Trans. R. Soc. B.

[196] Feng Y., Fu H., Zhang X., Liu S., Wei X. (2024). Lysosome Toxicities Induced by Nanoparticle Exposure and Related Mechanisms. Ecotoxicol. Environ. Saf..

[197] Guarnieri D., Sabella S., Muscetti O., Belli V., Malvindi M.A., Fusco S., De Luca E., Pompa P.P., Netti P.A. (2014). Transport across the Cell-Membrane Dictates Nanoparticle Fate and Toxicity: A New Paradigm in Nanotoxicology. Nanoscale.

[198] Isibor P.O., Sunday A.S., Buba A.B., Oyewole O.A. (2024). Mechanism of Nanoparticle Toxicity. Environmental Nanotoxicology: Combatting the Minute Contaminants.

[199] Zhang R., Qin X., Kong F., Chen P., Pan G. (2019). Improving Cellular Uptake of Therapeutic Entities through Interaction with Components of Cell Membrane. Drug Deliv..

[200] Wang H., Li G. (2018). Membrane Biophysics: New Insights and Methods.

[201] Jakutavičiūtė M., Ruzgys P., Tamošiūnas M., Maciulevičius M., Šatkauskas S. (2017). Physical Methods for Drug and Gene Delivery Through the Cell Plasma Membrane. Advances in Anatomy Embryology and Cell Biology.

[202] Kell D.B. (2016). How Drugs Pass through Biological Cell Membranes—A Paradigm Shift in Our Understanding?. Beilstein Magazine.

[203] Vikal A., Maurya R., Patel P., Das Kurmi B. (2024). Nano Revolution: Harnessing Nanoparticles to Combat Antibiotic-Resistant Bacterial Infections. Curr. Pharm. Des..

[204] Meliani A., Khelil F.Z.A., Nair S. (2023). The High Penetrability of Nanoparticles into Bacterial Membranes: A Key of a Potential Application. Postep. Mikrobiol..

[205] Kodaparthi A., Hameeda B., Bastipati S.B., Golla S. (2023). Bactericidal Effects: Microbial Nanoparticles as Next-Generation Antimicrobials. Environmental and Microbial Biotechnology.

[206] Busi S., Rajkumari J. (2019). Microbially Synthesized Nanoparticles as next Generation Antimicrobials: Scope and Applications. Nanoparticles in Pharmacotherapy.

[207] Ozdal M., Gurkok S. (2022). Recent Advances in Nanoparticles as Antibacterial Agent. ADMET DMPK.

[208] Mohammed E.H. (2023). Nanoparticles Antimicrobial Activity: A Mini Review. Iraqi J. Nat. Sci. Nanotechnol..

[209] Muñoz-López J., Lopes C.D., Batalha Í.L. (2025). Overview of Nanotherapeutics for Bacterial Infections. Nanotherapeutics for Infectious Diseases.

[210] Vargas E., Zhang F., Ben Hassine A., Ruiz-Valdepeñas Montiel V., Mundaca-Uribe R., Nandhakumar P., He P., Guo Z., Zhou Z., Fang R.H. (2022). Using Cell Membranes as Recognition Layers to Construct Ultrasensitive and Selective Bioelectronic Affinity Sensors. J. Am. Chem. Soc..

[211] Stapf H., Selbmann F., Joseph Y., Rahimi P. (2024). Membrane-Based NEMS/MEMS Biosensors. ACS Appl. Electron. Mater..

[212] Mo Y., Fei T. (2012). Nanoporous Membrane for Biosensing Applications. Nano Life.

[213] Sexton L.T., Horne L.P., Martin C.R. (2011). Biosensing with Nanopores and Nanotubes. Molecular-and Nano-Tubes.

[214] Emilsson G., Dahlin A.B. (2018). Nanopore Membranes for Separation and Sensing. Miniature Fluidic Devices for Rapid Biological Detection.

[215] Bahrami A., Doğan F., Japrung D., Albrecht T. (2012). Solid-State Nanopores for Biosensing with Submolecular Resolution. Biochem. Soc. Trans..

[216] Mokrushnikov P.V. (2020). A Mechanism of the Interaction of Metal Oxide Nanoparticles with Biological Membranes. Biophysics.

[217] Kręcisz M., Rybka J.D., Strugała A.J., Skalski B., Figlerowicz M., Kozak M., Giersig M. (2016). Interactions between Magnetic Nanoparticles and Model Lipid Bilayers—Fourier Transformed Infrared Spectroscopy (FTIR) Studies of the Molecular Basis of Nanotoxicity. J. Appl. Phys..

[218] Rodríguez-Hernández A.G., Vazquez-Duhalt R., Huerta-Saquero A. (2020). Nanoparticle-Plasma Membrane Interactions: Thermodynamics, Toxicity and Cellular Response. Curr. Med. Chem..

[219] Leroueil P.R., Berry S.A., Duthie K., Han G., Rotello V.M., McNerny D.Q., Baker J.R., Orr B.G., Banaszak Holl M.M. (2008). Wide Varieties of Cationic Nanoparticles Induce Defects in Supported Lipid Bilayers. Nano Lett..

[220] Jahandoost A., Dashti R., Houshmand M., Hosseini S.A. (2024). Utilizing Machine Learning and Molecular Dynamics for Enhanced Drug Delivery in Nanoparticle Systems. Sci. Rep..

